# Supplementary material for: A theoretical model of Polycomb/Trithorax action unites stable epigenetic memory and dynamic regulation
Source: Nat Commun. 2020 Sep 22;11:4782. doi: 10.1038/s41467-020-18507-4 (PMC7508846; doi:10.1038/s41467-020-18507-4)
Supplement: Supplementary file 1 — Supplementary Information [file 41467_2020_18507_MOESM1_ESM.pdf]

Supplementary material

A theoretical model of Polycomb/Trithorax action unites stable epigenetic memory and dynamic regulation

Reinig et al.

Supplementary Figures 1-10

Supplementary Tables 1 - 6

Supplementary Methods: Mathematical Modelling

Supplementary Discussion

Supplementary References

Supplementary Figures and Tables

Supplementary Figure 1. Coupling relationship between promoter and PRE
Related to main Figure 1.

(A, B) Two different coupling relationships are shown (model 1 and model 2). For each model, *Factor_p3* and *Factor_p4* are calculated according to the promoter state. Similarly, *Factor_p1* and *Factor_p2* are calculated according to the PRE/TRE state. *C* = coupling constant, defines coupling strength. Promoter: N_B = number of B sites, N_F = number of F sites, *s* = total number of sites**.** PRE/TRE: N_A = number of A nucleosomes, N_M = number of M nucleosomes, *S* = total number of nucleosomes. At the end of each iteration, the parameters *p1, p2, p3* and *p4* are multiplied by the values of *Factor_p1, Factor_p2, Factor_p3* and *Factor_p4* respectively. **(C, D)** The plots show the response of *Factor_p3* and *Factor_p4* (and thus of the parameters *p3* and *p4*) to promoter state state (left) and the response of *Factor_p1* and *Factor_p2* (and thus of the parameters *p1* and *p2*) to the PRE/TRE state, for the two coupling models shown in (A, B). Factors whose value is proportional to the extent of activation are shown in green, those whose value is proportional to the extent of silencing are shown in red**. (E, F)** Dependence of the two models on coupling strength and relationship between the models. The plots show ln(*Factor_p4/Factor_p3*) plotted against promoter state (left) and ln(*Factor p1/Factor p2*) plotted against PRE/TRE state (right). Different values of the coupling constant *C*, for model 1 (E) and model 2 (F) are shown. Values of *C* that give equivalent response in both models are shown. See Supplementary methods for details. **(G, H)** Parameter scans to evaluate the behaviour of model 1, model 2 and six models derived from each, are shown. The models were evaluated for their ability to mediate memory of silencing (calculated as in Supplementary Figure 2), memory of activation (calculated as in Supplementary Figure 3), and to produce the gradient pattern of the *eya1::GFP* transgene (calculated as described in the legend to Supplementary Figure 9). Yellow colour indicates good fit in each case. The PRE/TRE parameters (*p3, p4*) and *p5* were varied as shown. C_e in all simulations = 0. For memory of silencing and memory of activation, C_{im} = 8 for models 1 and 3 - 8; C_{im} = 4 for models 2 and 9 - 14. For the *eya* gradient, C_{im} = 0 for all models; C_{eya} = 2.5 for models 1 and 3 - 8; C_{eya} = 1.25 for models 1 and 9-14. The models differ in the parameters that are adjusted at each iteration as shown on the right of each set. For models 3 - 6, the coupling relationship for each parameter is identical to that used in the full model 1. Likewise, models 9 - 12 use the coupling relationship of model 2. Models 7 and 13 use the coupling relationship of models 1 and 2 respectively for all parameters, but allow all four parameters only to be reduced by the coupling, and not increased. Models 8 and 14 use the coupling relationship of models 1 and 2 respectively for all parameters, but allow all four parameters only to be increased by the coupling, and not reduced.

Supplementary Figure 1.

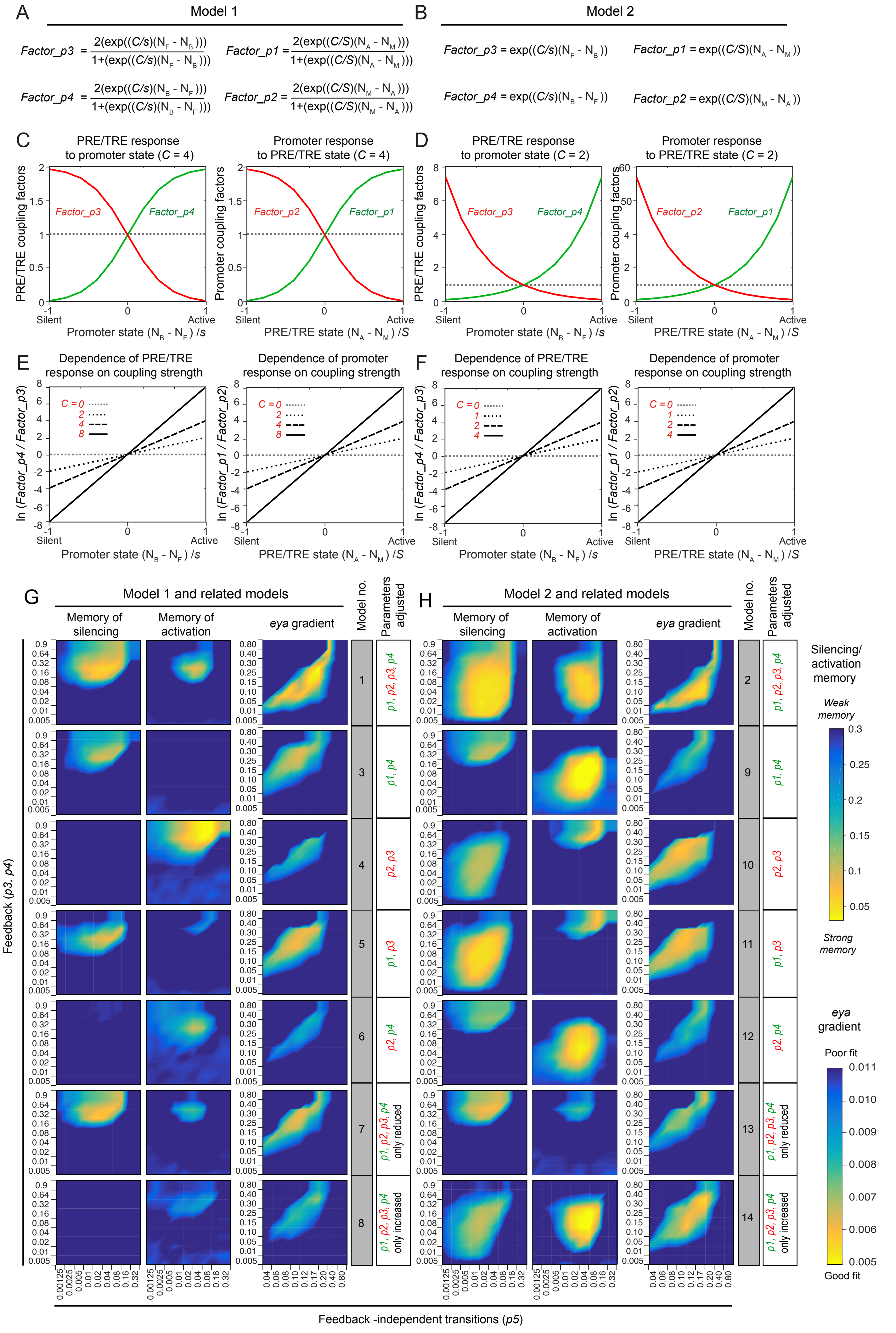

Supplementary Figure 2. Comparison of two models for memory of silencing

Related to main Figure 2.

(A) Simulated time courses of *Drosophila* development showing promoter and PRE/TRE activities over time for the first 7.5 hours of development, without coupling (left) and with coupling according to model 1 (middle) or model 2 (right) (for coupling relationships see Supplementary Figure 1). The models were implemented with input parameters and initial conditions as for Figure 2B, see also Supplementary Table 2.), with the exception of $C_{i,m}$ (coupling in initiation and maintenance phases) = 4 for model 1, $C_{i,m}$ = 2 for model 2. A: anterior; P: posterior. **(B)** Boxplots of promoter (scale: 0 to 1) and PRE/TRE (scale: -1 to +1) activities for each of the conditions and compartments shown in (A). Plots show data for promoter and PRE/TRE levels at 7h30 for 400 independent simulations. N_B: number of promoter sites in B configuration, *s*: total number of sites; N_A, N_M: number of PRE/TRE nucleosomes in A or M configurations respectively, *S*: total number of nucleosomes. Central mark on box plots: median; bottom and top edges of box: 25th and 75th percentiles, respectively. Whiskers extend to cover 99.3% of the data. Outliers are plotted as dots. **(C)** Calculation of silencing memory. Left: the promoter level is averaged over the last 10 minutes of the initiation phase in the absence of the PRE/TRE, for each simulation (1,…,n). This vector I (see also red box in panel A) gives the baseline pattern which must be maintained by the PRE/TRE. Right: the promoter level is averaged over the last 10 minutes of the maintenance phase in the presence of the PRE/TRE, for each simulation (1,…,n). This vector M (see also red boxes in panel A) gives the pattern that is maintained by the PRE/TRE. The extent of similarity between the vectors I and M indicates the strength of memory mediated by the PRE/TRE. For fitting, a single value silencing memory is calculated as the mean squared difference between I and M at each position as shown. Memory score = 1 - silencing memory. **(D)** Parameter scans for models 1 and 2. The input values of parameters (*p3, p4*) and *p5* were varied as shown, for different values of $C_{i,m}$ as indicated on the plot. C_e = 0 for all simulations. Black crosses on bottom panels indicate the parameter combinations used for the simulations shown in A. The colour scale shows silencing memory calculated as shown in panel (C), averaged over 200 independent simulations for each parameter combination. Yellow colour indicates the strongest memory. See also Figures 2 and Supplementary Figure 1.

Supplementary Figure 2

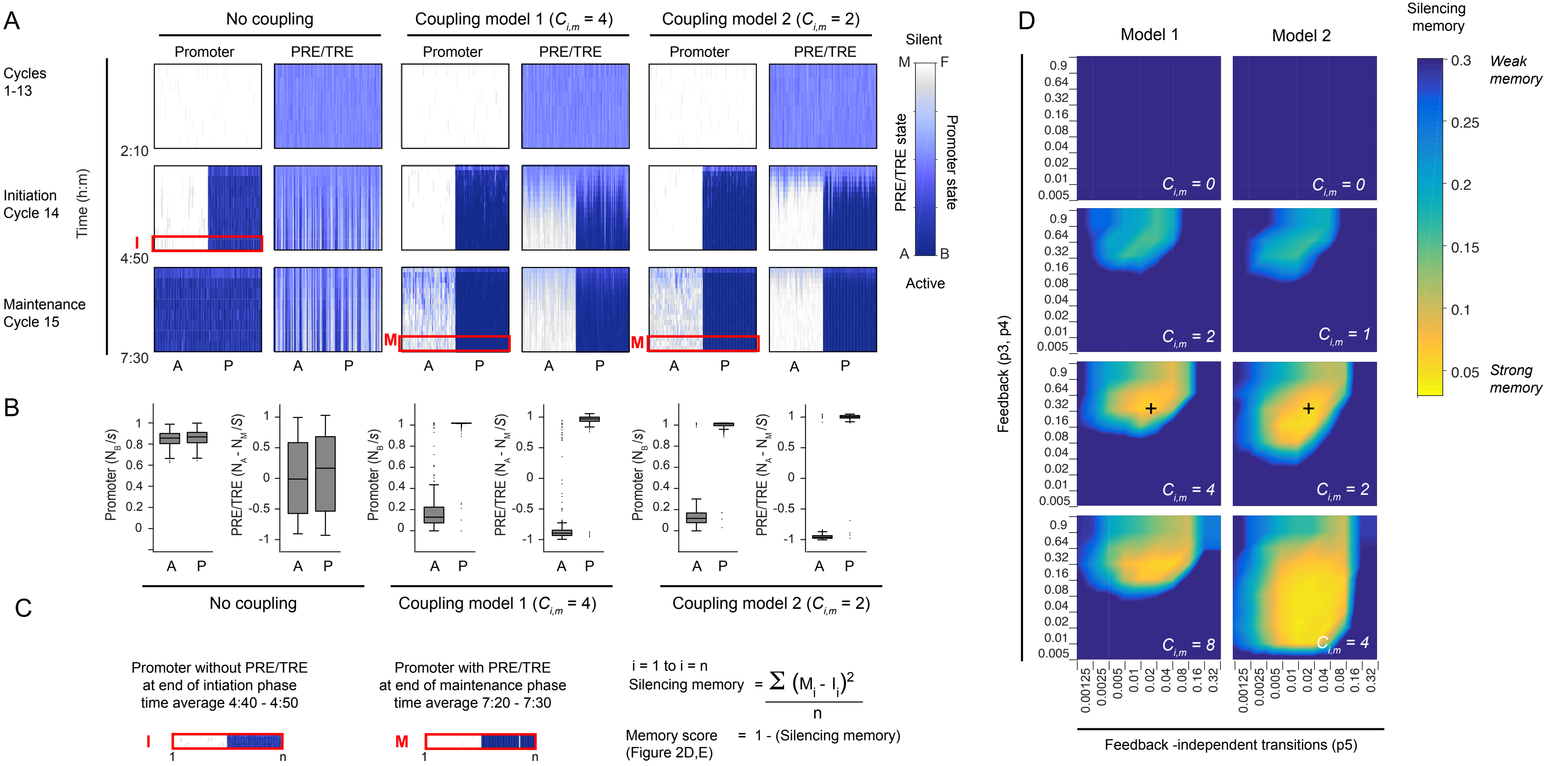

Supplementary Figure 3. Comparison of two models for memory of activation

Related to main Figure 3.

(A) Simulated time courses of *Drosophila* development showing promoter and PRE/TRE activities over time for the first 7.5 hours of development, without coupling (left) and with coupling according to model 1 (middle) or model 2 (right). In all simulations, a heat shock, indicated by Hs on the plot, was introduced during the first 60 minutes of the initiation phase (2h10 to 3h10), by increasing *p1* to 0.6. (*p1* input during cycles 1-13 = 0.001; *p1* input after heatshock = 0.06). All other input parameters and initial conditions as for Figure 3D, see also Supplementary Table 2.) C_e (coupling between PRE/TRE and promoter during cycles 1-13) = 0; $C_{i,m}$ (coupling in initiation and maintenance phases) = 4 for model 1, $C_{i,m}$ = 2 for model 2. For the plots shown in (A), each condition was simulated 100 times independently. **(B)** Boxplots of promoter (scale: 0 to 1) and PRE/TRE (scale: -1 to +1) activities for each of the conditions and compartments shown in (A). Plots show data for promoter and PRE/TRE levels at 7h30 for 400 independent simulations. N_B: number of promoter sites in B configuration, *s*: total number of sites; N_A, N_M: number of PRE/TRE nucleosomes in A or M configurations respectively, *S*: total number of nucleosomes. Boxplot parameters as in Supplementary Figure 2B. **(C)** Calculation of activation memory. Left: the promoter level is averaged over the last 10 minutes of the heat shock in the absence of the PRE/TRE, for each simulation (1,…,n). This vector H (see also red box in panel A) gives the heat-shock activated level of the promoter, which must be maintained by the PRE/TRE. Bottom: the promoter level is averaged over the last 10 minutes of the maintenance phase in the presence of the PRE/TRE, for each simulation (1,…,n). This vector M (see also red boxes in panel A) gives the promoter level that is maintained by the PRE/TRE. The extent of similarity between the vectors H and M indicates the strength of memory of activation mediated by the PRE/TRE. For fitting, a single value activation memory is calculated as the mean squared difference between H and M at each position as shown. **(D)** Parameter scans for models 1 and 2. The parameters (*p3, p4*), and *p5* were varied as shown, for different values of $C_{i,m}$ as indicated on the plot. C_e = 0 for all simulations. Black crosses on bottom panels indicate the parameter combinations used for the simulations shown in A. The colour scale shows activation memory calculated as shown in panel (C), over 200 independent simulations. Yellow colour indicates the strongest memory. See also Figures 3 and Supplementary Figure 1.

Supplementary Figure 3

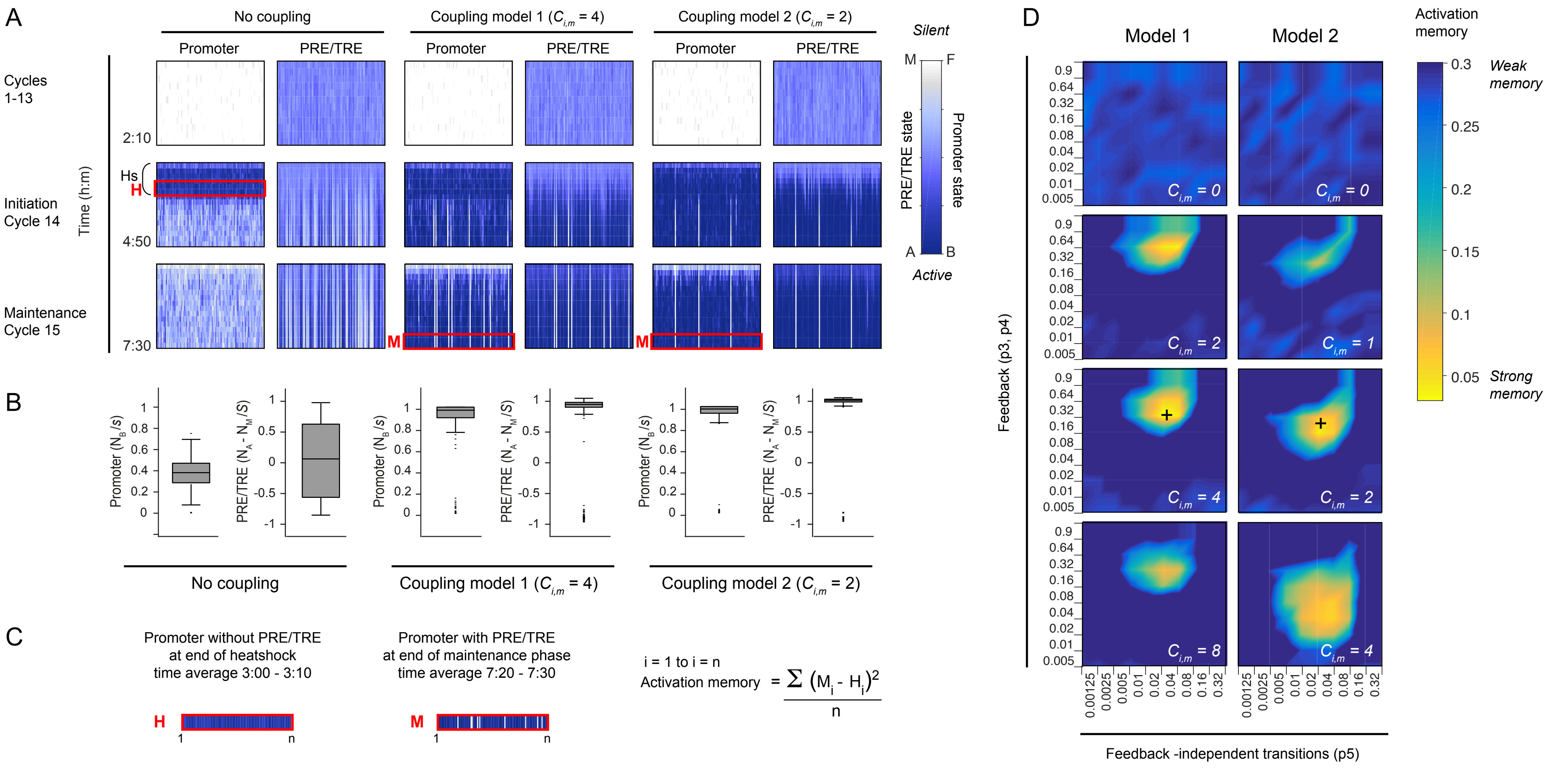

Supplementary Figure 4. Antibody specificity. Related to main Figure 4.
Histone H3 tail peptides were spotted onto nitrocellulose membrane in the quantities indicated, and incubated with each of the primary antibodies as indicated above the figure. Signals were visualized by enhanced chemiluminescence (ECL). See also Figures 4 and Supplementary Figure 5.

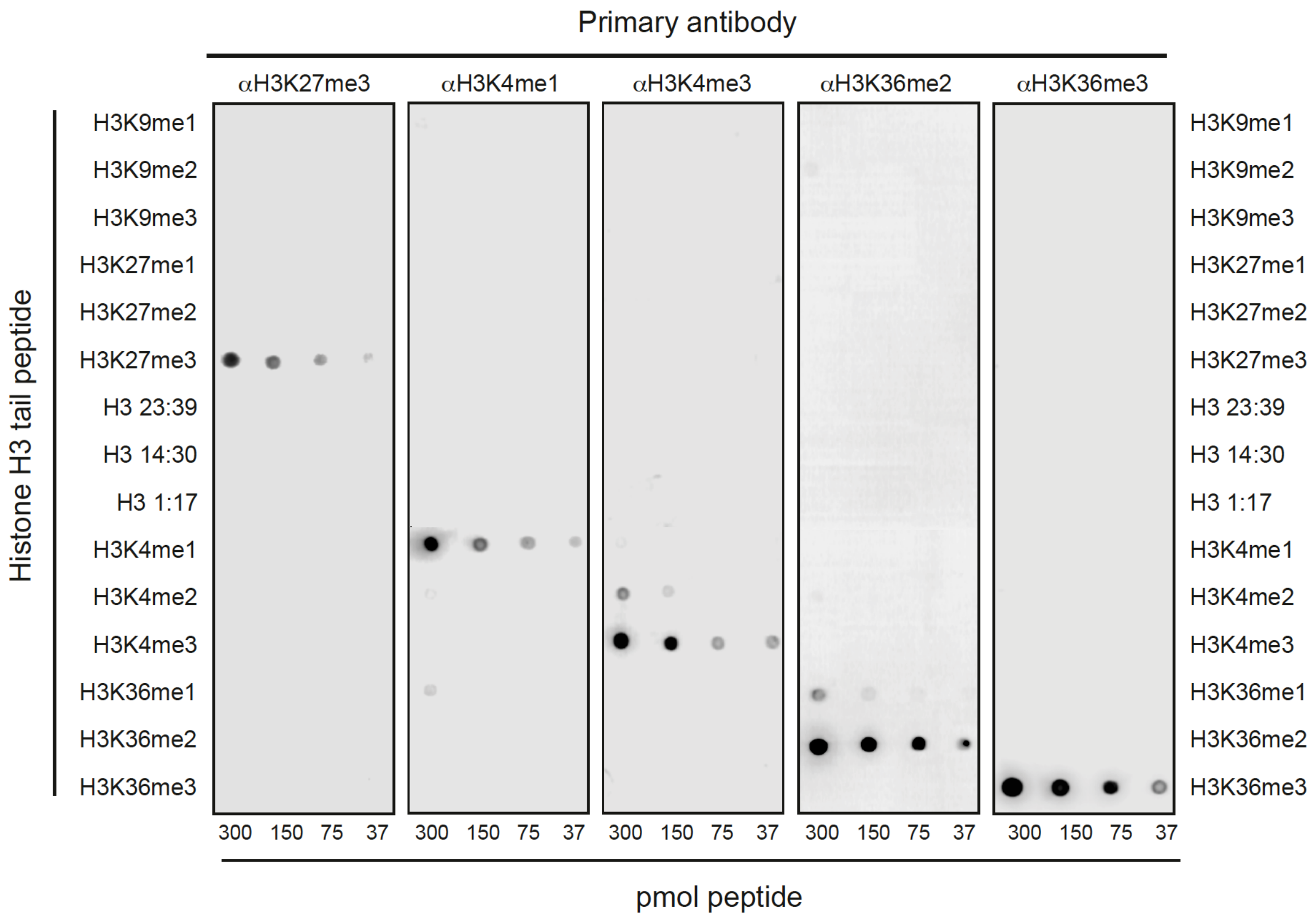

Supplementary Figure 5. Analysis of PC protein and histone methylation during development

Related to main Figure 4.

(A-D) Embryos were fixed and double stained with antibodies against unmodified histone H3 (green, left panels) and Polycomb (A), H3K36me3 (B), H3K4me1 (C) or H3K4me3 (D) (magenta, middle panels). Merged images are shown in the right panels. Embryos were staged according to morphology, as indicated. Three slides per antibody were prepared, typically containing 50 to 100 embryos, of which five to 10 were at the required stage and all showed similar staining for a given stage. Mitotic cells are indicated at stages 7 and 9 (m). Scale bar represents 75 μm and is the same for all panels. White boxes at stages 5 and 6 indicate pole cells (p), which first become apparent at stage 5. A'- D' show 3 X zoom of boxed areas. 'p' indicates that the protein or mark of interest is detectable in pole cell nuclei. Where the pole cells were detectable but no nuclear staining was seen, the pole cells are indicated with a white arrowhead (e.g., B' Stage 5 and D'). See also Figure 4.

Supplementary Figure 5

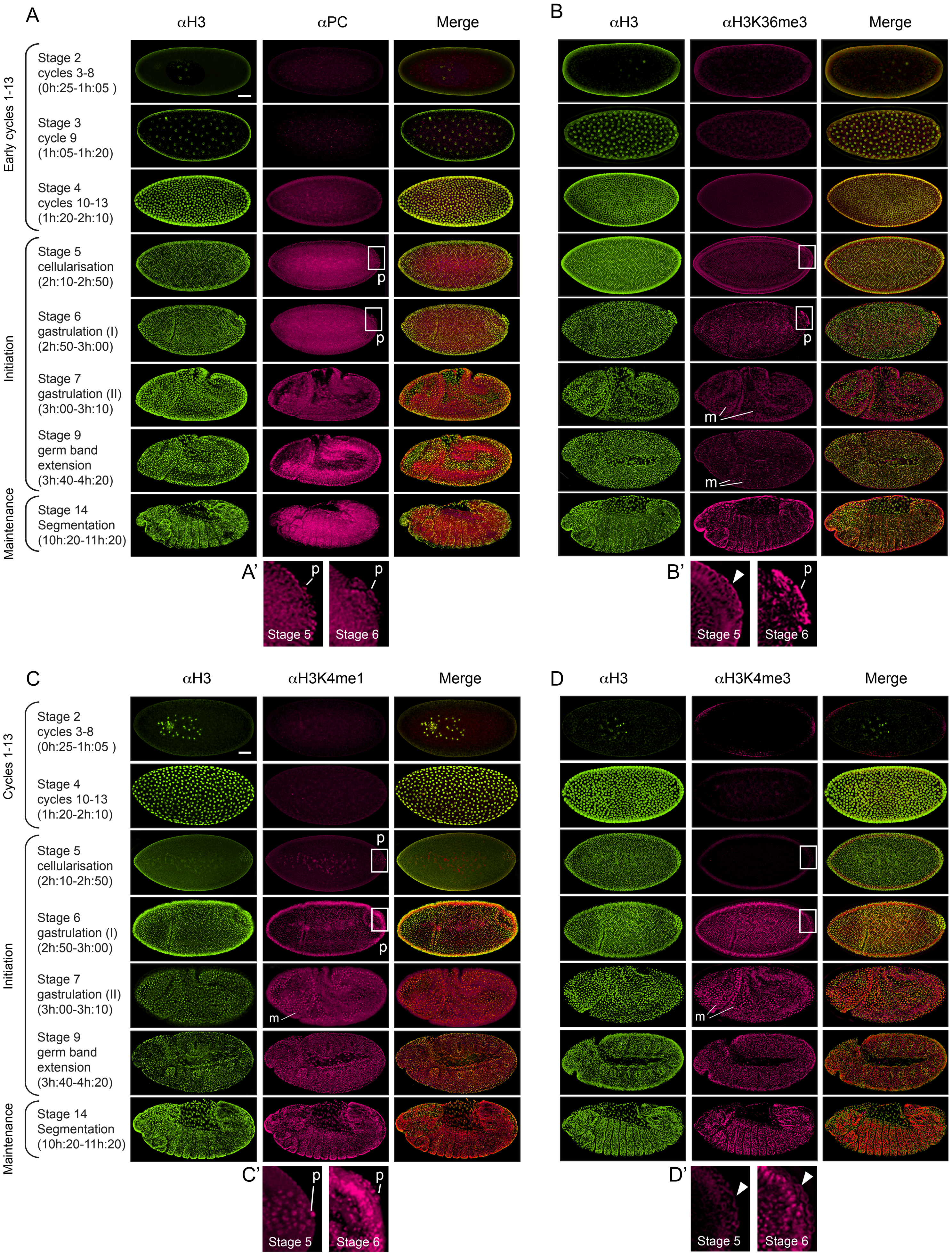

Supplementary Figure 6. Comparison of published data with model predictions, ChIP quality control

Related to main Figure 4.

(A) Simulated time course of accumulation of M nucleosomes as a proportion of total nucleosomes (*S*) in the array over developmental time, averaged over 1,000 individual simulations. Model 1 was used with parameters as in Figure 2B,C (no coupling between PRE/TRE and promoter). **(B)** Summary of time points addressed in this study (Reinig et al.), in [1] (Li et al., 2014) and in [2] (Bonnet et al., 2019). **(C)** Correlation between pairs of independent ChIP data series in this study is shown. For each time point, two independent chromatin preparations were made as described in methods. From each chromatin preparation one input and two IPs for each of the antibodies shown in Figure 4E was performed (PC, H3, H3K27me3, H3K36me2, H3K36me3, H3K4me1, H3K4me3). Each IP and input sample was processed as described in methods and subjected to qPCR in a 5- fold dilution series. For a given antibody and a given dilution, pairs of Ct values from one ChIP from each of the two chromatin samples are plotted as a single point on the scatter plot (black dots). The inputs from the two samples are similarly plotted (red dots). Input was diluted 100 – fold before qPCR analysis to ensure Ct values in the same range as the ChIP samples (see methods). The slope of the fitted line in each case is close to 1, showing that the whole data series from the two independent chromatin samples are similar in terms of absolute Ct values. Comparison of the second ChIPs gave similar results. The R^2 value shows a high correlation in each case (>= 0.94) indicating low variation between replicates.

Supplementary Figure 6

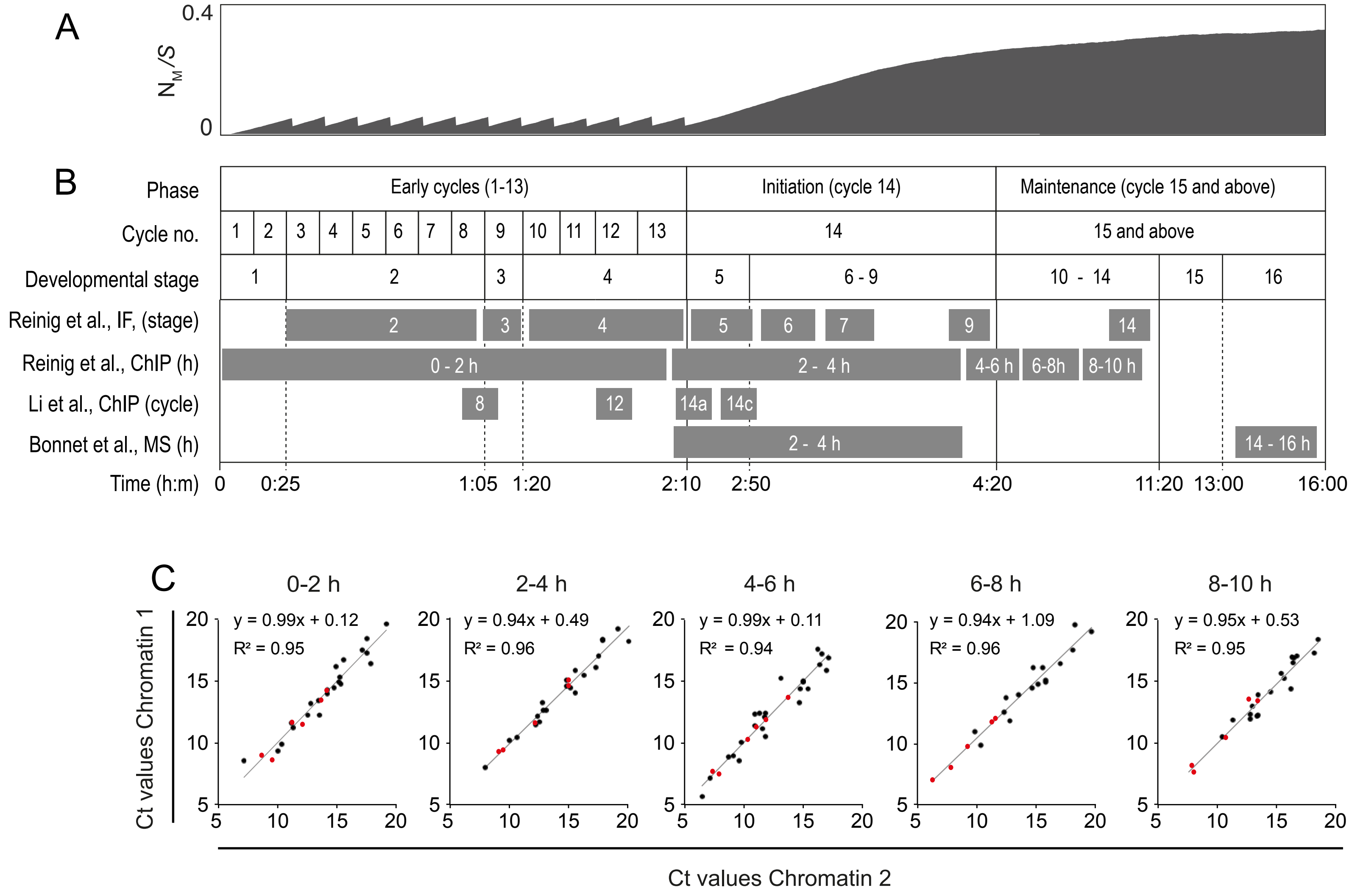

Supplementary Figure 7. Comparison of *eya* and *bxd* PREs

Related to main Figure 5.

(A) Motif composition of the *eya* PRE/TRE (coordinates 6546210-6544711, *D.mel* genome version R5.28). **(B)** Motif composition of the *bxd* PRE/TRE (coordinates 12590916-12589364, *D.mel* genome version R5.28). Motifs are defined as in the key to panel A. Underlined symbols indicate consecutive runs of a motif. Nucleotides are named according to the UIPAC code. **(C)** Motif counts in the *eya* PRE/TRE (black) and *bxd* PRE/TRE (grey). **(D)** The genomic sequence of the intronic *eya* PRE/TRE is shown, with colour coded binding sites for GAF (green), PHO (red) and Zeste (yellow). In the *eya1::GFP* ΔPHO transgene shown in panels S and T, the eight PHO binding sites were mutated to the following sequences (selected to avoid creating a new activating site). Site 1) CGCTA; site 2) CATGT; site 3) ACGAA; site 4) ACCAT; site 5) GTTAT; site 6) GTATG; site 7) CACTA; site 8) CACTA.

Supplementary Figure 7

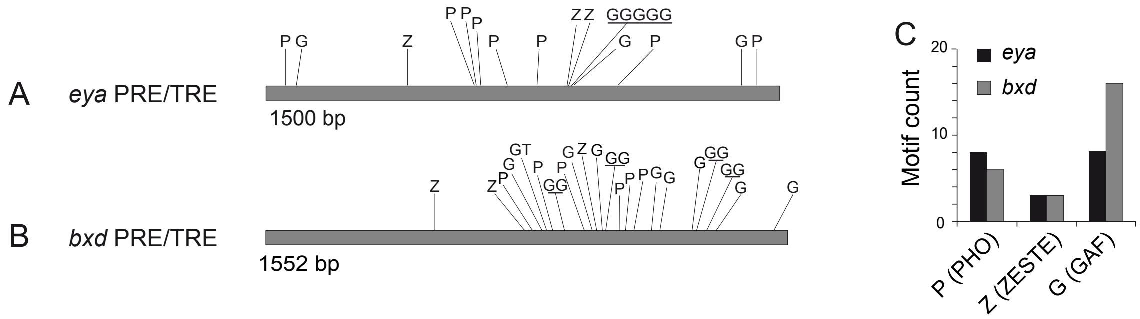

D *eya* PRE/TRE *Chr 2L:6546243-6544711*

```
CAAACCTGACTCCTGGTGCTCTCAATCATATTTTCCACATCAAGTGCGCATAATTGCTATCCTTTAATGCGTTCCTCTAGCTCCGAAATGGCCAAAATGGTAG
CAGTTGATCGTGAGAGATAAGCTCACTGTACAGTGAACTGTAAGAAATCAAAGATGCTACTACGCATTTGCCCAGTGCTGATCCTTTAAATGCAGGACACAAT
GCAGACAGTAGTCAAAAGGGAATTTTCAGCTTAAGTGCGCACAAAGGGCCACAGTCAGATATCCAGATATCCTGTCGATCGAGAAGGAATCATTTGACCAGAA
ATAAAACCGCAAAAAGGATCAGTTTGAGAAGGCACTAGGTCAAGGGCTGTAACTCAATGGAAGGTAGAGATCTTTAGATCGGAATTTCTGCTGAATCCCGCTT
TGATTGACAGTTGTACGCTCATTTCCTATCCTTCTCTGCTTCTCTGTTAAGTGAGTTCCATTCCACATAATCGTGCCCTCGTTGCCAGACAGCTCCCTCACAA
CTCCGGCGTAACTTGAGCAAACTTTTCGGGCAAAGCAATTTGCGATGTGTTTGTCTCGTTGCCAAGGCAAACAGAACACGGCTTTTTCCTGCCAGCGGCCATC
CAAAATGGCTACCGTCGATGGCGGCCGGAAGGAAGAAGCAGAAGAAGGCAGAAAGCCAAAAACGTCAGAGATACAAAAGCGTCAACTGCCGCCATTTTTTTTC
CGGGTATCTATTTGACAGCATCGGAGACAACACGAAAATCTCATAAATGGGAATGCAAAAAAGGACGAGCCATTAGCAAATAATTTCAGCGAAAAACATTTTA
TTCGGCATTGTTTGGCGAGCTGGAGGGAAAAAGACGGCTACACACGGTGAGTGAGTGAGAGAGAGAGAGAGAGAGGGAGAGGACAGGCAAATGCCGTGAGGAT
AATTTCATTGAAATTTGAGTAGTCAGAAAGGAGCAGCAGAGAATGTACAAAAGGACAACGCCGTGCAGATGCCTTGCGCTTTTCGTTTTGCCATTAAAACAAC
AAACAATGCAGCCAGGAGCGGGACAGGATCAGGGGTTTCAACTGGGGGTCCTTCAGCACCTCATCCCCCTCATCACCGTTTCGGGATTTTGCGCTACCCGCAG
GACTTCTCGCTCTGCGGGTTCCCAACGATAGCGAGCATTTCAATTCGTTTCTCGTTTGTGGCCGGGCTGCTCCATTTCAGCCACGCCCTGGGAATGTCCCAAA
AATTAAAATTAAATGCAAGGGGGTGGCCTATTCGCTTGAAGCTCATCACATTATCCCCCCAGGCTCAAATGCCTGGCGACATTTTTACAAAATAATCTGTGAA
TCTGTGAGTCCAGGCATTACATCTCTCATCTCCCAGCTCTTAATCCGCATTGTCCTTCGGCTTAAATGGCAAATGTTTGTCTGGGGCGTGGCACAGGAAGTGC
AACCATCTCTGTCTGGAAGGGATATACATACACCTACATACCTGTTCCCGTTCGAAGTCGAGGAATTTATTTGGGTTTCTGCCACTTCAACAAATGAACTTGT
TTGTTGTTACTC
```

Supplementary Figure 8. Modeling the third instar eye disc and fitting to experimental data
Related to main Figure 6.

(A) Model of *eya* activation in 3rd instar larval eye disc comprises multiple time course simulations for cells at different positions in the disc along the anterior-posterior axis. During the third instar, the morphogenetic furrow (MF, red) moves across the eye disc from posterior to anterior. The *eya* gene (here, the model promoter, white indicates maximum activity of 1.0) is activated shortly prior to the passage of the MF and decays over time afterwards. See Supplementary methods for details. **(B)** To generate a snapshot of the model disc at mid-third instar, a row of cells along the anterior posterior axis was constructed from the time course for each position at 96h of development. 20 such rows of cells were compiled to generate the image shown. **(C)** To generate an average profile of the promoter output across 5 simulated discs, the promoter output at each of these positions was averaged for all 100 rows. For details, see Supplementary methods. **(D)** The *eya1::GFP* reporter construct contains regulatory sequences of the *eya* gene, driving a *GFP* reporter gene. Variants were generated with and without the PRE/TRE (see Figure 5). Grey bar indicates *in situ* probe. **(E)** Relative quantification of *GFP* mRNA levels by in *in situ* hybridisation in 3rd instar larval eye discs. Top: merge of DAPI (blue) and *GFP* mRNA (green). Bottom: Maximum intensity image of *GFP* mRNA signal. Scale bar, 100μm for both images. Six - 10 discs for each transgene gave similar results. Line scans along the anterior posterior axis, perpendicular to the MF, were performed for 6-10 discs. See supplementary methods for detail on normalisation and scaling. **(F)** The average profile for a given transgenic construct was plotted**. (G)** The experimental data without the PRE/TRE (see Figure 5) was used to fit the shape of the gradient determined by the *eya* enhancer alone. For this the model was used without coupling to the PRE/TRE. *p2* (TF dissociation) was fixed at 0.1, and *p1* (TF association) was varied as described in Supplementary methods, to achieve the best fit between the model and experimental profiles shown in (C) and (F). **(H)** The model was fitted to the experimental data with the PRE/TRE by introducing coupling between the model promoter and PRE/TRE (see Figure 5). The *p1* values determined in (G) were fixed, and the model profile was fitted to the experimental data by varying the PRE/TRE parameters *C,(p3, p4)* and *p5*. See supplementary methods and Supplementary Figure 9 for details.

Supplementary Figure 8

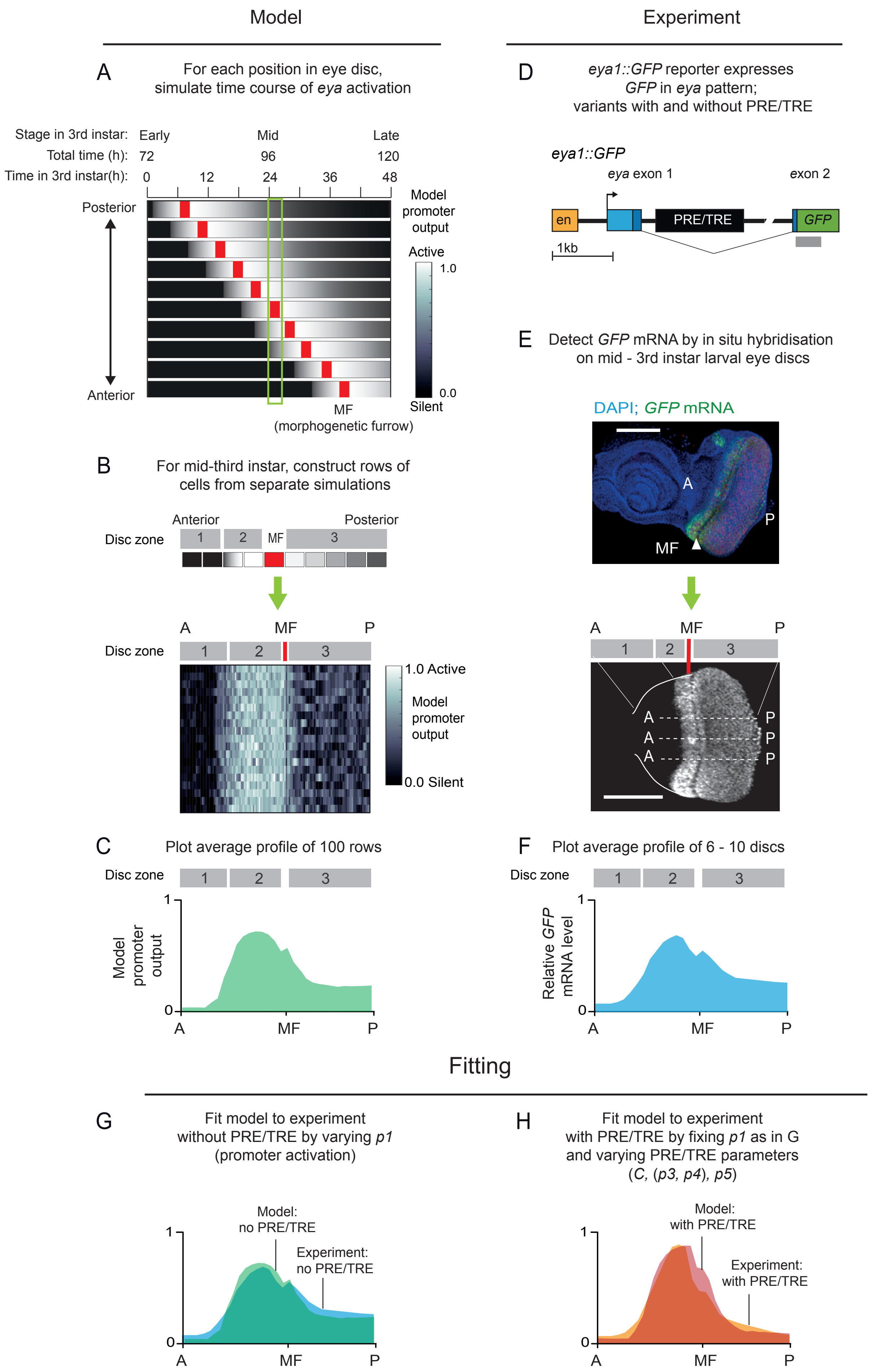

Supplementary Figure 9. Comparison of two models for *eya1::GFP* PRE/TRE transgene with different PRE/TREs

Related to main Figure 6.

(A) Model eye disc. The eye disc was modeled as described in Supplementary methods and Supplementary Figure 8. The output of the model promoter without PRE/TRE (left) and coupled to the *eya* PRE/TRE according to model 1 (middle) or model 2 (right) is shown. PRE/TRE parameters C_{eya}, *(p3,p4)* and *p5* that gave optimal fits to experimental data for the *eya* PRE/TRE (Figure 5) are shown above the plots. In all simulations, $C_{i,m} = 0$. **(B)** Profiles of model promoter output with and without the *eya* PRE/TRE, averaged over 100 independent simulations. **(C)** Model eye discs with parameters that best fit the experimental data for the *bxd* PRE/TRE (see Figure 5). **(D)** Profiles averaged over 100 independent simulations. **(E-F)** Parameter scans for models 1 and 2, in which the model promoter output was fitted to the experimental gradient shape of the *eya1::GFP* reporter gene with the *eya* PRE/TRE (E) or with the *bxd* PRE/TRE (F). The colour scale shows goodness of fit (yellow indicates best fit), calculated as mean squared difference between the model and the experiment at each position along the anterior- posterior axis of the disc for each parameter combination. Black crosses on (E, F) indicate the parameter combinations used for the simulations shown in (A, C). Note that the optimum parameter ranges for the *eya* and *bxd* PRE/TREs are mutually exclusive. See Supplementary methods for details. **(G)** Variegation was scored as follows: For each parameter combination, 100 independent simulations were performed and profiles were generated. The standard deviation (SD) from the mean of these 100 simulations was scored at each position along the profile. SD was then averaged for the whole profile, giving a single value for each parameter combination. A high SD is caused by variegation (i.e. a mixture of active and silent states, as shown in panel C.). A lower SD indicates that the promoter output is more similar from one simulation to the next (e.g., as in panel A). Yellow indicates high SD and thus high variegation. Black crosses on (F, G) indicate the parameter combinations used for the simulations shown in C, which fulfil the criteria of both strong variegation and a good fit to the experimental *bxd* profile. **(H, I)** The plots show the response of *Factor_p3* and *Factor_p4* (and thus of the parameters *p3* and *p4*) to promoter state state (left) and the response of *Factor_p1* and *Factor_p2* (and thus of the parameters *p1* and *p2*) to the PRE/TRE state, for the two coupling models (H: model 1 with C = 2.5; I: model 2 with C = 1.25, see also Supplementary Figure 1A, B). Factors whose value is proportional to the extent of activation are shown in green, those whose value is proportional to the extent of silencing are shown in red.

Supplementary Figure 9

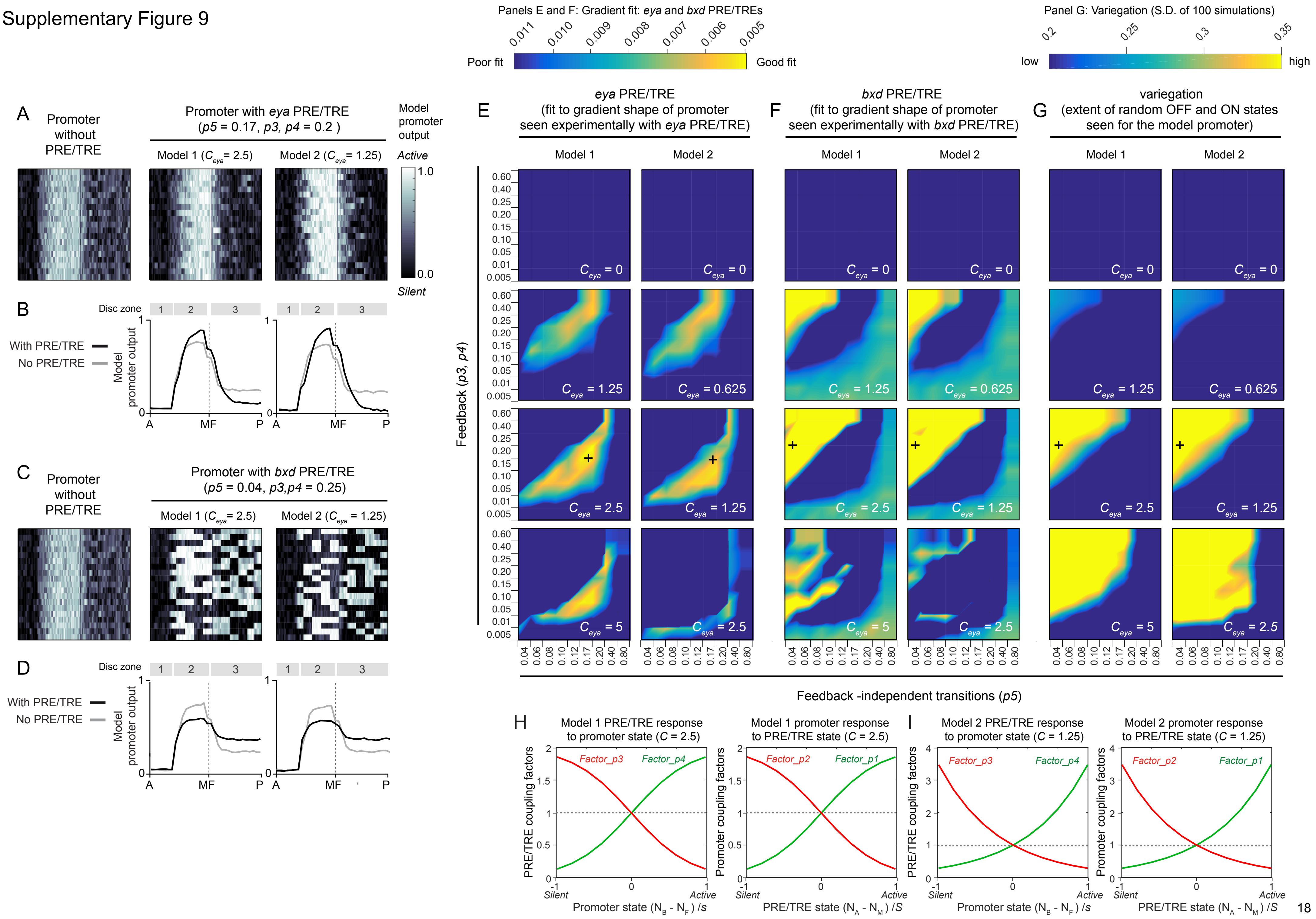

Supplementary Figure 10. Comparison of one-PRE/TRE and two-PRE/TRE models
Related to main Figure 6.
(A-D) Four models are shown, which differ in whether they contain one PRE/TRE (A and C) or two (B and D). **(E-H)** Parameter scans for models A to D, in which the model promoter output was fitted to the experimental gradient shape of the *eya1::GFP* reporter gene with the *eya* PRE/TRE (E) or with the *bxd* PRE/TRE (G,H). In (F), the model was fitted to data from the *eya1::GFP* reporter gene both with and without the PRE/TRE (see Supplementary Methods for details). For each parameter combination, 100 independent simulations were performed and the averaged profile was fitted to the experimental data shown in Figure 6F (*eya* PRE/TRE) or 6H (*bxd* PRE/TRE). The colour scale shows goodness of fit (yellow indicates best fit), calculated as mean squared difference between the model and the experiment at each position along the anterior- posterior axis of the disc for each parameter combination. Scales for E, G, H: 0.005 - 0.011; scale for F, 0.007 - 0.01. Note that the optimum parameter ranges for the *eya* and *bxd* PRE/TRE models are mutually exclusive. Black crosses indicate the parameter combinations used for the simulations shown in (J-U). **(I)** Variegation was scored as described in the legend to Supplementary Figure 9. Scale 0.15 - 0.35. **(J-Q)** The output of the model promoter for different models as indicated above each figure, are shown. **(R-U)** Profiles of model promoter output for each model, averaged over 100 independent simulations. The parameter combinations used for each simulation are indicated above each panel.

Supplementary Figure 10

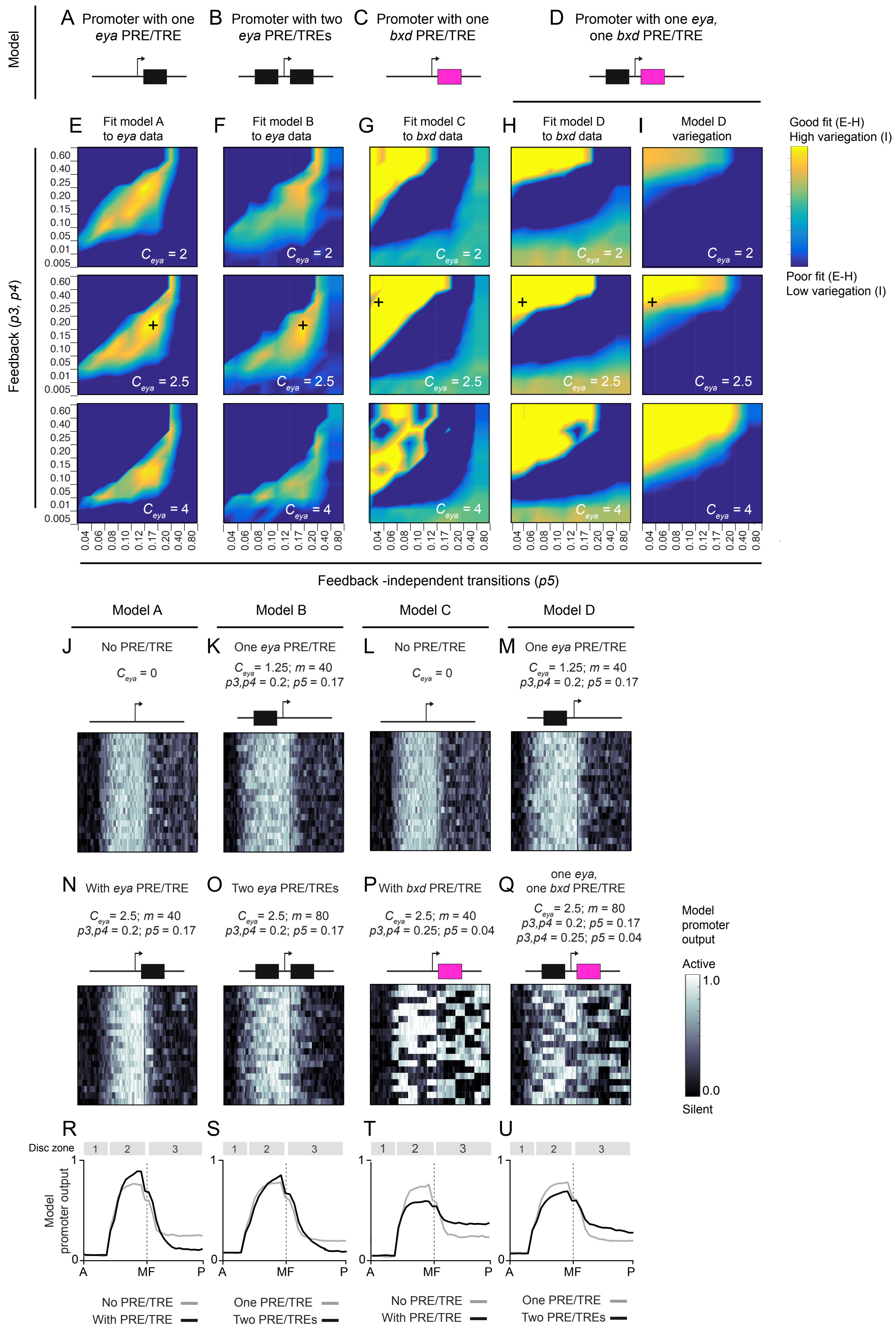

Supplementary Tables

Supplementary Table 1. Developmental timing in *Drosophila* and in the model

The table shows the timing of developmental events in *Drosophila* at 25°C. Length of each cell cycle is given where known. The corresponding cell cycle times and total simulation times as implemented in the model are shown. On the right of the table the relevant features of developmental transcriptional regulation that are recapitulated by the model are shown. The dotted line between cycle 14 and 15 indicates that cycle 14 is of variable length depending on cell type. For simplicity in the model, the length of cycle 14 is fixed at 2h40.

Supplementary Table 1. Developmental timing in *Drosophila* and in the model

Drosophila development					Model features			Common features of development and model		
**Developmental events**	**Time at end of stage (h:m)**	**Stage**	**Cycle**	**Cycle length**	**Time at end of stage (h:m)**	**Cycle**	**Cycle length**	**Enhancer activity**	**PcG/ TrxG**	**Phase**
Cleavage	00:25	1	1-2	8 min		1-13	10 min	No transcription	On PRE/ TREs	Cycles 1-13
	01:05	2	3-8							
	01:20	3	9							
	02:10	4	10-13	8-20 min	02:10			Segmentation genes		
Cellularisation	02:50	5	14	1h:20-3h		14	2h:40	*Hox* enhancers		Initiation
Gastrulation	03:00	6								
	03:10	7								
Germ band extension	03:40	8								
	04:20	9			04:50					
	05:20	10	15 and above	Not well studied		15	2h:40	*Hox* repressors disappear	Mutant effect	Maintenance
	07:20	11			07:30					
Germ band retraction	09:20	12				16 and above	Can vary in model			
	10:20	13								
Head involution, dorsal closure, dorsal segmentation	11:20	14								
	13:00	15								
Full segmentation, muscle differentation	16:00	16								
	22:00	17			22:00					
Eye disc proliferation	49:00	1st instar	Eye disc: 1-7	ca. 10h		Eye disc: 1-7	10h	Larval enhancers		
	72:00	2nd instar								
	92:00	mid 3rd instar			92:00					
Eye disc 2nd mitotic wave	Variable	mid to end of 3rd instar	Eye disc: 8	ca 8h	100:00	Eye disc: 8	8h	*eya* activation		
Eye disc post-mitotic	120:00		Post mitotic	variable	120:00	Post mitotic	20h	*eya* decay		
Adult eye	216:00	Pupa-adult	Post mitotic	n.a	216:00	Post mitotic	n.a			

Supplementary Table 2. Parameters

Related to main Figures 1- 7

(A) List of parameter values used in different versions of the model. Where parameters used for models 1 and 2 differ, the model 2 parameters are shown in brackets. **(B)** Meaning of parameter values in developmental time. Selected parameter values from (A) are shown. The parameter value gives the probability of an event occurring at each iteration (one iteration = 12 sec). The probability of events per minute and per sweep are given. One sweep is the number of iterations equal to the number of nucleosomes in the PRE/TRE. i.e., for a PRE/TRE of size 40, one sweep is 40 iterations = 8 mins.

Supplementary Table 2. Parameters.
A) Parameter values used in the models.

Parameter	Definition	Figure 2: anterior	Figure 2: posterior	Figure 3	Figure7F: *eya* PRE/TRE	Figure7G: *bxd* PRE/TRE
p1e	TF binding (cycles 1-13)	0.001		0.001	0.001	
p1hs	TF binding during heatshock	n/a		0.6	n/a	
p1i	TF binding in initiation phase (cycle 14)	0.001	0.6	0.06	0.01	
p1m	TF binding in maintenance phase (cycle 15)	0.6		0.06	0.01	
p1disc	TF binding in 3rd instar (larval disc cycle 1-7)	n/a		0.06	0.01	
p1eya1	TF binding during eya activation (larval disc cycle 8)	n/a		n.a	0.6	
p1eya2	TF binding during eya decay (larval disc after cycle 8)	n/a		n.a	0.25	
p2	TF dissociation	0.1		0.1	0.1	
p3	Recruited conversion to M	0.25		0.25	0.2	0.25
p3m	Recruited conversion to M in PcG mutant	0.001		n/a	n/a	
p4	Recruited conversion to A	0.25		0.25	0.2	0.25
p5	Recruitment independent conversion	0.04		0.04	0.17	0.04
C_e	Coupling between PRE/TRE and promoter (cycles 1-13)	0		0	0	
$C_{i.m}$	Coupling between PRE/TRE and promoter from cycle 14 to 3rd instar larva. Model 2 values in brackets.	4 (2)		4 (2)	0	
C_{eya}	Coupling between PRE/TRE and promoter after onset of eya expression. Model 2 values in brackets.	n/a		n/a	2.5 (1.25)	
h_a	Hill coefficient for fitting activation of *eya* enhancer ahead of morphogenetic furrow (larval disc cycle 8).	n/a			3	
K_a	Constant (*eya* activation)	n/a			0.4	
t_a	Time factor for Hill equation (*eya* activation)	n/a			600	
h_d	Hill coefficient for fitting decay of *eya* enhancer behind morphogenetic furrow (larval disc after cyc 8).	n/a			2	
K_d	Constant (*eya* decay)	n/a			0.85	
t_d	Time factor for Hill equation (*eya* decay)	n/a			100	
S	PRE/TRE size (number of nucleosomes)	40				
s	Promoter size (number of binding sites)	10				

(B) Meaning of model parameters in developmental time

	iterations = 12 sec	sweeps = 8 mins
1 hour	300	7.5
12 hours	3600	90
1 day	7200	180

Parameter value (probability of an event occuring at each iteration)	events min^{-1}	events $sweep^{-1}$
0.001	0.005	0.04
0.01	0.05	0.4
0.04	0.2	1.6
0.06	0.3	2.4
0.1	0.5	4
0.17	0.85	6.8
0.25	1.25	10
0.32	1.6	12.8
0.6	3	24

Supplementary Table 3. Peptides.

Peptides were purchased from Jpt Innovative Peptide Solutions in unbiotinylated form.

Peptide name	Peptide sequence
H3 1:17	H-ARTKQTARKSTGGKAPR-NH2
H3 14:30	H-KAPRKQLATKAARKSAP-NH2
H3 23:39	H- KAARKSAPATGGVKKPH-NH2
H3 1:17 K4me1	H-ART-Lys(Me1)-QTARKSTGGKAPR-NH2
H3 1:17 K4me2	H-ART-Lys(Me2)-QTARKSTGGKAPR-NH2
H3 1:17 K4me3	H-ART-Lys(Me3)-QTARKSTGGKAPR-NH2
H3 1:17 K9me1	H-ARTKQTAR-Lys(Me1)-STGGKAPR-NH2
H3 1:17 K9me2	H-ARTKQTAR-Lys(Me2)-STGGKAPR-NH2
H3 1:17 K9me3	H-ARTKQTAR-Lys(Me3)-STGGKAPR-NH2
H3 23:39 K27me1	H-KAAR-Lys(Me1)-SAPATGGVKKPH-NH2
H3 23:39 K27me2	H-KAAR-Lys(Me2)-SAPATGGVKKPH-NH2
H3 23:39 K27me3	H-KAAR-Lys(Me3)-SAPATGGVKKPH-NH2
H3 23:39 K36me1	H-KAARKSAPATGGVLys(Me1)-KPH-NH2
H3 23:39 K36me2	H-KAARKSAPATGGVLys(Me2)-KPH-NH2
H3 23:39 K36me3	H-KAARKSAPATGGVLys(Me3)-KPH-NH2

Supplementary Table 4. Primary antibodies.

For dot blot, primary antibodies were used at 1:1000 dilution. For immunofluorescence, primary antibodies were diluted 1:5000, with the exception of αH3, which was diluted 1:1000. For ChIP, IPs were performed using 50μg DNA in 300μl volume with 4.5μg of each primary antibody. For in situ hybridization, the primary antibody was used at 1:500 dilution. For antibodies against histone modifications, validation is shown in Supplementary Figure 4. For validation of the anti histone H3 antibody see https://www.activemotif.com/catalog/details/39763

Name	Supplier	Catalog / lot no.	Application
Mouse anti Histone H3	Active Motif	39763 / 5217020, Mab clone 301	ChIP, IF
Rabbit anti H3K4me1	Abcam	ab8895 / gr271478-1	ChIP, IF, dot blot
Rabbit anti H3K4me3	Abcam	ab8580 / gr240214-3	ChIP, IF, dot blot
Rabbit anti H3K27me3	Active Motif	39155 / 31814017	ChIP, IF, dot blot
Rabbit anti H3K36me2	Abcam	ab9049 / gr266894-1	ChIP, IF, dot blot
Rabbit anti H3K36me3	Abcam	ab9050 / gr249065-1	ChIP, IF, dot blot
Mouse anti Fluorescein	Roche	11426320, Mab clone 001	In situ hybridisation

Supplementary Table 5. Secondary antibodies.

For dot blot and immunofluorescence, secondary antibodies were used at 1:1000 dilution. For in situ hybridization, the secondary antibody was used at 1:100 dilution.

Name	Supplier	Catalog / lot no.	Application
Goat anti Rabbit IgG, Alexa Fluor 555 conjugate	Thermo Fisher Scientific	A21429 / 1715464	Immuno-fluorescence
Goat anti Mouse IgG, Alexa Fluor 488 conjugate	Thermo Fisher Scientific	A11029 / 1705900	Immuno-fluorescence
Goat anti Rabbit IgG, Horse Radish Peroxidase (HRP) conjugate	Life Technologies	A16104 / 42-28-042114	Dot- blot
Goat anti Mouse IgG, Horse Radish Peroxidase (HRP) conjugate	Life Technologies	A16072 / 46-137-05115	Dot- blot
Goat anti Mouse IgG, Horse Radish Peroxidase (HRP) conjugate	Invitrogen	T-20912	In situ hybridisation

Supplementary Table 6. DNA oligonucleotides.

A) PCR primers and templates

Application	Product / primer name	Template	primer sequence fw 5'-3'	primer sequence rev 5'-3'
Amplification of ChIP material; genome-wide qPCR	24mer	ChIP material from staged embryos	AGAAGCTTGAATTCGAGCAGTCAG	
	20mer		CTGCTCGAATTCAAGCTTCT	

B) eya1::GFP cloning and in situ probes

construct	product	template	primer sequence fw 5'-3'	primer sequence rev 5'-3'
in situ probes	eya exon 1.1	genomic DNA	GAAAGCGGACCACCATAAAC	AGTTTTGATAGCACGGCACA
	GFP	TurboGFP plasmid (Evrogen)	GCCATGGAGATCGAGTGC	GGTGTTGCTGTGATCCTCCT
eya1::GFP	*eya* fragment A	genomic DNA	GCGCACTTAAGTAGCTTAAACAGAC	ATTTAGACCAGGAGACAACAATGAG
	eya fragment B	genomic DNA	TGTTTTGAGGGACTTCTTTAGGG	ATGTGTATCCGTGTGGTCTGTCT
	Turbo GFP (fragment C)	TurboGFP plasmid (Evrogen)	TTTCAGGTTAAACGTGAGAGCGACGAGAGC	ATGAGTTTGGACAAACCACAAC
	eya fragment B for fusion	Fragment B in PCRII	TGTTTTGAGGGACTTCTTTAGGG	GCTCTCGTCGCTCTCACGTTTAACCTGAAA
	Fusion product BC	TurboGFP (fragment C); *eya* fragment B for fusion	TGTTTTGAGGGACTTCTTTAGGG	ATGAGTTTGGACAAACCACAAC
eya1::GFP deletion constructs	*eya1::GFPΔen* left	*eya1::GFP*	GAGCACGTGTGTGTGCTTCT	AACACTTTAAGGATAAAATCGCATACGGCCAGTTTCGTCTCC
	eya1::GFPΔen right	*eya1::GFP*	GGAGACGAAACTGGCCGTATGCGATTTTATCCTTAAAGTGTT	GGGGAAACACAGGCACATAA
	eya1::GFPΔen left right fusion	*eya1::GFPΔen* left; *eya1::GFPΔen* right	GAGCACGTGTGTGTGCTTCT	GGGGAAACACAGGCACATAA
	eya1::GFPΔPRE/TRE left	*eya1::GFP*	ACCATTCACACCACCAAAAA	GAGTAAACAAACAAACAAGTTCATTTGAGCACCAGGAGTCAGGTTTG
	eya1::GFPΔPRE/TRE right	*eya1::GFP*	CAAACCTGACTCCTGGTGCTCAAATGAACTTGTTTGTTTGTTTACTC	CAAAAAGCAGGTCCTTCGAG
	eya1::GFPΔPRE/TRE left right fusion	*REGFPΔPRE/TRE* left; *REGFPΔPRE/TRE* right	ACCATTCACACCACCAAAAA	CAAAAAGCAGGTCCTTCGAG
PRE/TRE replacement construct	*bxd* PRE/TRE	genomic DNA	atgctagcctaggGCTTGTCGAATTCAAAAAGAATTA	ttgagctcggtaccatCTCTCTTTCGTTTTCCGCTTCT

Supplementary Methods: Mathematical modelling

See also [https://github.com/Ringrose546/Beyond_memory_V2]

1) Model components: A promoter, a PRE/TRE, and regulated coupling between them

1.1) Promoter- PRE/TRE interactions

During the initiation phase of early *Drosophila* development (2h-5h), *Hox* gene expression patterns are established by transcriptional activators and repressors that bind to embryonic enhancers. Later in development, during the maintenance phase (after 5h) the positional information given by transcription factors is lost, and the correct *Hox* expression patterns are maintained by Polycomb/Trithorax Response Elements (PRE/TREs) (reviewed in [3]). Thus during the initiation phase, the *Hox* enhancer and promoter must instruct the PRE/TRE, and during the maintenance phase, the PRE/TRE must instruct the enhancer and promoter. We have modelled these interactions using minimal stochastic models for a promoter and a PRE/TRE, and introduced coupling between them. Known cell cycle lengths for the different stages of *Drosophila* development were introduced into the simulations. The model parameters were fitted to recapitulate three different experimental observations, namely memory of silencing [4] memory of activation [5] and modification of an expression gradient (this work).

1.2) Promoter model

The promoter is modelled as an array of DNA binding sites for a transcription factor (total number of sites, *s*. Figure 1A). Each site can be either free (F) or bound (B). The promoter state is given by the proportion of sites that are in the B configuration. We do not distinguish between enhancers and promoters in the model: the state of the promoter is assumed to be a direct readout of the state of its associated enhancer. We also do not explicitly distinguish between transcriptional activators and repressors: the promoter output is given by the proportion of sites that are in the F or B configuration at any given time, with F contributing to repression and B contributing to activation. We assume in the model that promoter output is directly proportional to the amount of transcript and protein produced, i.e. we do not include terms for RNA stability and translational efficiency, since these are unknown in the cases we study here. In the version of the model used here, we assume that there is no cooperativity between binding events at different sites across the array. Thus the output of the promoter is a simple analogue response to the relative probabilities of binding and unbinding (*p1* and *p2*, Figure 1B). Cooperativity has not been studied for the

promoters and enhancers we address here, but could easily be included to apply the model to other cases where cooperativity is known to play a role in promoter activation or repression.

1.3) PRE/TRE model

The PRE/TRE is modelled essentially as described in [6]. The PRE/TRE consists of an array of nucleosomes (total number: *S*), each of which can be in a silent (M) neutral (U) or active (A) configuration (Figure 1A). The PRE/TRE state is given by the proportion of nucleosomes that are in the A or M configurations (in the active state, A nucleosomes dominate, in the silent state, M nucleosomes dominate). For a given nucleosome, each of the A and M configurations represents all histone modifications and bound proteins that contribute to activation or silencing. Although each nucleosome contains two copies of each histone, the stoichiometry of bound proteins per nucleosome is unknown. For this reason we use whole nucleosomes as the minimum unit, as in [6], and not half nucleosomes as in [7]. For a given nucleosome, the A and M configurations are mutually exclusive, and conversion between A and M has to pass through the intermediate U (Figure 1B). Feedback in the model from each of the A and M configurations renders the intermediate U configuration unstable and the A and M configurations stable (Figure 1B). Models for chromatin - mediated epigenetic memory based on this simple bistable system or adaptations of it have been applied successfully to mating type switching in yeast [6], vernalisation in *Arabidopsis* [7] and DNA methylation in mammals [8].

Given the complexity of Polycomb and Trithorax regulation, what is the evidence that this minimal model is appropriate for the questions addressed here? Polycomb/Trithorax regulation involves multiple chromatin modifications, enzymatic reactions that add or remove modifications, and binding events that are independent of chromatin modifications [3,9]. Recently, over 70 publications on Polycomb/Trithorax biochemistry in flies and vertebrates have been integrated into a comprehensive mathematical model, showing that the system is robustly bistable, preferring to adopt either extreme active or extreme silent states [10]. Thus the simple bistable PRE/TRE model we use here is an appropriate simplification.

It has recently been proposed that the activating signal that opposes silent histone marks is transcription itself [11]. The PRE/TRE model that we use here does not exclude the possibility that the A state has a contribution from transcription, which would be consistent with observations of non-coding transcription through PRE/TREs that can in some contexts, switch the PRE/TRE to an

active state [9,12]. However we note that ample feedbacks exist between activating proteins and modifications that are independent of transcription itself [10], thus the model does not explicitly require transcription at the PRE/TRE to oppose silencing.

Instead, the A and M nucleosome configurations in the model comprise all modifications and bound proteins (or processes) that contribute to activation and silencing respectively. U refers to nucleosomes that lack these modifications and bound proteins (i.e. H3K27, H3K4 and H3K36 are unmodified and PcG and TrxG proteins are not bound). The biochemistry of the PcG/TrxG system is described in detail in [10], and references therein. Here we summarise. The cooperative interactions that contribute to silencing are described in the model by the feedback that converts U to M nucleosomes in a manner that depends on the proportion of existing M nucleosomes in the system (Figure 1B, *p3*, short curved red arrow). Likewise the cooperative interactions that contribute to activation are described in the model by the feedback that converts U to A nucleosomes in a manner that depends on the proportion of existing A nucleosomes in the system (Figure 1B, *p4*, short curved green arrow). The antagonistic interactions are represented in the model by the feedbacks that convert each extreme nucleosome configuration (A or M) to U depending on the abundance of nucleosomes in the opposite configuration (Figure 1B, *p3* and *p4*, long curved arrows).

Since very few of the enzymatic reaction rates are known, and the effect of feedback stimulations has not been quantified, the parameters *p3* and *p4* were kept equal to each other in the model, and thus they represent one feedback parameter. We note however, that evidence exists for PRE/TREs being biased towards activation or silencing depending on developmental stage [12], PRE/TRE sequence and genomic location [13]. Thus for specific cases *p3* and *p4* can be varied with respect to one another, though we do not exploit this flexibility in this work. Furthermore either of these two parameters can be reduced whilst the other is unchanged, in order to simulate the effects of *PcG* mutations (reduction of *p3*, see Figure 2B), or *trxG* mutations (reduction of *p4*).

In addition the model allows conversions between A, U and M nucleosomes that are independent of feedback, described in the current model by the parameter *p5*, (Figure 1B, straight black arrows) which was varied to fit the model to different data. This includes histone exchange and random noisy conversions as described previously [6,7] but also includes specific conversions that do not

require a previously existing modification for a modifying enzyme to be recruited, such as direct recruitment by DNA binding proteins [14].

In the model, a nucleosome can be influenced by any other nucleosome in the array, and not only by its nearest neighbour. Thus within the PRE/TRE, local distance effects are not taken into account (the PRE/TRE is approximately 8 kb if 40 nucleosomes are spaced at 200bp). Spreading of histone marks from a nucleation site has been shown to be important in several studies [7,15,16]). However, we reason that the assumption we use in the model is valid because we model the PRE/TRE as a single entity, and we are interested in the effect of coupling it to the promoter. Thus the question we are interested in is not: how does a modification spread through the PRE? but rather to what extent is the PRE/TRE state dependent on and communicated to the promoter? The coupling relationships described below enable this to be addressed.

1.4) Coupling the promoter and the PRE/TRE

The unique feature of the model described here in comparison to previous models of epigenetic regulation is that transcription is considered explicitly and distinctly from chromatin states. In previous models, chromatin state has been used as a proxy for transcription [6,7], or transcription itself has been considered as the state opposing silent chromatin modifications (thus assuming that active chromatin states do not exist independently of transcription itself [11]). Here we model the PRE/TRE and the promoter as separate units, each of which can be in an active or silent state independently of the other. The active state of the PRE/TRE may involve non- coding transcription [9,12] consistent with the proposal of [11], but the important feature is that the active state of the coding gene promoter and that of the PRE/TRE are independent of each other. We then allow regulated coupling between them (Figure 1C). Although we found that regulated coupling was essential to recapitulate the experimental data, the molecular nature of this coupling is unknown. Thus we tested 14 different models for coupling to understand to what extent the results are dependent on the mathematical description used (Supplementary Figure 1). This identified two models (which we named 1 and 2) that gave optimum results. The 14 different models for coupling have in common that they allow adjustment of PRE/TRE parameters in response to the promoter, and adjustment of promoter parameters in response to the PRE/TRE (Figure 1C). In essence the effect of coupling is to ensure that the more silent or active the promoter, the more silent or active the PRE/TRE, and

vice versa. The difference between the models is in the functions used to describe this relationship, and the choice of parameters that they affect (Supplementary Figure 1).

The implementation of coupling during simulation is summarised in Figure 1C, shown in detail in Supplementary Figure 1, and described below (see simulations: the core model). The coupling factors *Factor_p1, Factor_p2, Factor_p3* and *Factor_p4* are calculated depending on the state of the PRE/TRE and promoter according to the equations shown in Supplementary Figure 1A and B for model 1 or model 2. The effect of PRE/TRE and promoter state on the values of each coupling factor is shown in Supplementary Figure 1 C,D for one value of coupling strength, (*C*). However, the coupling strength can be changed to change the steepness of the system response (Supplementary Figure 1E and F). The coupling factors are multiplied by the values of the parameters *p1*, *p2*, *p3* or *p4* at each iteration (see Figure 1C). Models 1 and 2 differ in the extent to which parameter values are amplified or suppressed. Model 1 favours suppression (Supplementary Figure 1C), whereas model 2 favours amplification (Supplementary Figure 1D). Importantly however, the quotient of pairs of opposing factors (*Factor_p4/Factor_p3* or *Factor_p1/Factor_p2*) is identical for a given promoter or PRE/TRE state for both models at equivalent values of *C*. (*C* (model1) = 2**C* (model 2), see Supplementary Figure 1E, F).

We found that models 1 and 2 gave similar but not identical model behaviours in three tests (memory of silencing, memory of activation and eye disc gradient, Supplementary Figure 1G,H; see sections 4.1 – 4.3 below for more details on these tests). Models 1 and 2 are compared for each data set throughout the paper, in Supplementary Figures 2, 3, and 9. The results of the two models are very similar, with similar optimum parameter combinations for each test. However, model 2 is more robust than model 1 in two tests, giving good results over a wider range of parameter values for memory of silencing (Supplementary Figure 2D) and memory of activation (Supplementary Figure 3D). Conversely, model 2 is somewhat less robust than model 1 for the eye disc gradient (Supplementary Figure 9E). The results can be understood as a higher requirement for parameter amplification (model 2) for memory of silencing and activation, but a higher requirement for parameter suppression (model 1) for generation of the eye disc gradient. For simplicity, results of model 1 are shown in the main figures but in all cases model 2 gave virtually identical results for the parameter combinations shown (see Supplementary Figures 2A,B, 3A,B and 9A-D).

Analysis of simpler models showed that it was necessary to adjust all four parameters (*p1, p2, p3* and *p4*) in order to achieve optimum performance in all three tests (Supplementary Figure 1G, H). Models in which only two parameters were adjusted (one for the PRE/TRE and one for the promoter) were suboptimal in one or more tests (see models 3-6 and 9-12, Supplementary Figure 1G, H). Models 1 and 2 adjust the four parameters by both reducing or amplifying their values, depending on PRE/TRE and promoter state. We next asked whether a simplified version of this self- reinforcing system would be sufficient, in which the values of *p1, p2, p3* and *p4* were only reduced, and not amplified (Supplementary Figure 1G, H, models 7 and 13). This analysis showed that whilst memory of silencing was still quite effective for both models under this constraint, the memory of activation was lost for both models, and model 13 was unable to reproduce the eye disc gradient. Conversely, models 8 and 14, in which the four parameter values were amplified but not reduced, were both impaired in silencing (Supplementary Figure 1G, H). Model 8 was also unable to give memory of silencing or the *eya* gradient for any parameter combination, whereas model 14 performed well in these two tests. We conclude that models 1 and 2 are the only ones of those tested that give optimal results in all three tests.

We assumed that coupling in both directions (from PRE/TRE to promoter and from promoter to PRE/TRE) is the same at a given coupling strength. We reason that coupling is very likely caused by molecular mechanisms that apply equally to both elements, once they come into proximity with each other by looping, spreading or pairing. Thus it would be difficult to justify a model in which one element affected the other but not *vice versa*. Mechanisms for coupling that do apply only in one direction may in fact exist (e.g., directional non-coding transcription in *cis*, or RNA mediated switching mechanisms that occur in *trans*). These could easily be implemented in the model. However since such mechanisms have not been described for the elements we study here, we have limited the models to the simple case in which coupling affects both elements simultaneously. This assumption was fully adequate to explain the observed data.

2) Developmental changes in cell cycle length: *in vivo* and in the model

2.1) Embryonic divisions

The length of the cell cycle changes extensively during *Drosophila* development and is mostly well characterised. A summary is given in Supplementary Table 1. The first 13 nuclear divisions are synchronous, and take place in a syncytium without cytokinesis. Each division lasts between 8 and 16 minutes at 25°C, increasing in length as development proceeds [17,18]. From the 14th cycle

onwards (starting at 2h:10), cellularisation takes place and the divisions become globally asynchronous. Starting approximately one hour into the 14th interphase, mitotic domains of locally synchronised cells become visible, which occupy specific positions on the embryo surface. The 14th cell cycle lasts between 1h20 and 3h, depending on the mitotic domain. A mitotic domain atlas mapping each domain and its timing in detail is given in [18]. The 15th division cycle is less well characterised. Some cells never divide again, whereas others (in particular those giving rise to the larval imaginal discs) undergo several more divisions.

2.2) Eye imaginal discs

Of interest for this study are the cells that give rise to the eye imaginal discs and eventually the adult eye. The eye is derived from the eye-antennal disc, which is formed at stage 12 (approx. 9h, see Supplementary Table 1) from 20 cells that are designated during earlier stages [19]. During the first to third larval instar stages the disc proliferates to give a structure consisting of about 2,000 cells (http://www.sdbonline.org/sites/fly/aimorph/eye2.htm). The length of these proliferative cell cycles has not been studied in detail. For the purposes of this study, we estimated each cycle to be on average 10 hours (7 divisions are required to produce at least 2,000 cells from 20 cells in the time window of 1st to mid 3rd instar which is approximately 70 hours, see Supplementary Table 1). This estimate agrees well with reported estimates of the doubling time of 12h for proliferating cells in the wing disc [20]. During the third instar larval stage, a further wave of synchronised mitosis takes place, passing across the eye disc from posterior to anterior, behind the morphogenetic furrow. Cells ahead of the morphogenetic furrow arrest in G1 for 6-8h, then enter mitosis after the passage of the furrow [20,21]. We estimate that this cell cycle lasts approximately 8h (eye disc cycle 8 in Supplementary Table 1, see also *eya* gradient below). After this final mitosis, cells differentiate to give rise to the photoreceptors in the eye ommatidia and do not divide again [19].

2.3) Modelling cell cycles

In the model, the cell cycle lengths described above were adapted as shown in Supplementary Table 1, so that the simulated time course resembled the developmental timing of *Drosophila* as closely as possible. A number of simplifications were introduced to facilitate implementation in the model: for example the initial nuclear divisions were averaged to 10 mins each, and cycles 14 and 15 (variable *in vivo*) were set to 2h40 each. For the eye disc, the cell cycle lengths shown in Supplementary Table 1 were used. (See *eya* gradient below for details). Importantly, all time course simulations are run starting from the first nuclear division in the embryo, and including the

full number and length of cell cycles up to the time point at which the results were analysed, so that the total developmental time in the simulation reflects the developmental time *in vivo*.

In the model, we included cell numbers and identities that are relevant for the cells of interest in each experiment: All simulations of somatic tissues include the first 13 early divisions. For memory of silencing, in which experiments examine *hox* gene expression in anterior and posterior compartments, we simulate a representative sample of anterior and posterior cells (typically between 50 and 400 depending on the purpose of the simulation). For experiments that relate to eye development we model each eye with 20 independent simulations under identical conditions, each representing a single eye precursor cell and its descendants (because the eye disc is formed from 20 cells that are designated during earlier stages). To evaluate average system behaviour a larger number of simulations was performed.

To model cell cycles for the promoter, all sites were set to F at the end of each cell cycle. This is based on the observation that many transcription factors dissociate from mitotic chromatin [22] and that transcription is actively and globally repressed during mitosis [23,24]. For the PRE/TRE, cell division was modelled as described previously (Dodd et al., 2007), by setting each nucleosomes to U with a probability of 0.5, at the end of each cell cycle. Thus on average, half of the nucleosomes are set to U at the end of each cell cycle. This is based on the observation that parental histones and their modifications are partitioned randomly to the two daughter chromosomes after replication, and that new incoming histones do not carry PcG and TrxG dependent modifications. Chromatin maturation during the next cell cycle is required to re-establish the full pattern of modifications (reviewed in [3]).

This model introduces several simplifications, whose effect is to reduce the complexity of the model, but that may be refined as more data becomes available in future. First, we did not model all daughter cells, but only one of the two daughters at each replication. Second, the stages of replication (S phase) and mitosis (M phase) occur simultaneously in the model - thus we omit G2 phase. Since it is not known whether transcription of PRE/TRE regulated genes continues during G2 phase, the separation of S and M phases would have introduced several unknown parameters into the model. Third, transcription ceases globally during mitosis [23,24], some transcription factors do not dissociate from mitotic chromatin, instead remaining bound and bookmarking genes for accelerated post mitotic activation [25]. Thus the complete reduction of all promoter sites to F may

not be appropriate for bookmarked genes. However bookmarking has not been demonstrated for the genes we addressed in this study, and thus we modelled them assuming total dissociation. We note that the model can easily be adapted to simulate bookmarking by simply allowing a proportion of promoter sites to remain bound after each cell cycle.

Finally, the simulation of cell cycle disruptions for the PRE/TRE is based on the assumption that all parental modifications are passed to daughter chromosomes after replication, resulting in 50% of marks on average, being passed to each daughter. This has been reported in several studies [26,27] but questioned by others [28]. If parental histone marks do not survive replication, then the probability of each nucleosome being wiped to U in the model at replication would be greater than 0.5. On the other hand, there is evidence that PcG and TrxG proteins remain associated with replicating chromatin [28-30]. Thus not only histone modifications but also the proteins that catalyse them may be passed to new chromatin. If PcG and TrxG quantities are not limiting then this implies that more than 50% of nucleosomes may carry memory across the replication fork. If so then the probability of each nucleosome being wiped to U in the model would be less than 0.5. However since the quantities discussed above are unknown, we chose to implement the model based on the simple assumption that 50% of nucleosomes are inherited on average to each daughter strand at each cell cycle. We note that the model can easily be adapted to simulate different degrees of memory by simply adjusting the probability of wiping to U at each cycle. In summary, the important aspects are that relative cell cycle lengths vary during development, and that the promoter and PRE/TRE must both recover from disruption. These features are faithfully recapitulated in the model, and the model was fully adequate to explain the observed data.

3) Simulations: the core model

The model was implemented in Matlab version R2015b (8.6.0). Code is available on request. The core system is simulated in 17 steps. One round of steps 1-17 constitutes one iteration, which is equivalent to 12 seconds in developmental time at 25°C (Supplementary Table 2B). This time window was fixed empirically. We found that 12 seconds per iteration gives sufficient time resolution in the early cycles for the system to undergo several iterations per cell cycle, but is not so finely grained that the entire model takes too long to run the longer simulations (e.g. over several days of developmental time for the larval simulations). For different cell cycle lengths, the appropriate number of iterations is performed before moving to steps 18 and 19 (DNA replication and mitosis). After replication, a single daughter chromosome is taken to the next iteration. In all

simulations the promoter consists of 10 sites and the PRE/TRE consists of 40 nucleosomes, determined by fitting the model to different data (see below). One sweep of the system is the number of iterations of steps 1-17 equal to the number of nucleosomes (= 40 x 12 sec, = 8 mins). Since both the promoter and the PRE/TRE are updated at each iteration, the promoter is swept four times for each PRE/TRE sweep (i.e. every 2 mins). See Supplementary Table 2B for parameter values and their meaning in developmental time.

Initial conditions

All simulations start at cycle 1 of embryogenesis. All nucleosomes in the PRE/TRE are set to U unless otherwise stated. All sites in the promoter are set to F. The values of the coupling factors *Factor_p3* and *Factor_p4* are set to 1 for the first iteration. Input values are set for *p1* (promoter binding), *p2* (promoter unbinding), *p3* (PRE/TRE recruited conversion to M), *p4* (PRE/TRE recruited conversion to A) and *p5* (PRE/TRE recruitment – independent conversions) (see Supplementary Table 2A for input values).

Modify PRE/TRE

1. The value of *p3co* and *p4co* are calculated: *p3co* = *p3* x *Factor_p3*; *p4co* = *p4* x *Factor_p4*.
2. A nucleosome n1 to be modified is chosen at random.
3. A second nucleosome n2 is chosen at random.
4. *Recruited conversion:* Depending on the configuration of n2 (A, U or M), a conversion of n1 towards this configuration is attempted:
5. If n2 is U, n1 is not changed.
6. If n2 is M, n1 is changed one step towards M with probability equal to *p3co*.
7. If n2 is A, n1 is changed one step towards A with probability equal to *p4co*
8. If n2 and n1 are in the same configuration, n1 is not changed.
9. *Recruitment - independent conversion:* The modified or unmodified n1 (from steps 5-8) is changed one step towards the adjacent configuration with probability equal to *p5*. If n1 is A or M, it is converted to U. If n1 is U it is changed to A with probability of 0.5 and to M with probability of 0.5.
10. The PRE/TRE state is evaluated by counting the number of nucleosomes in A, U and M configurations.
11. The values of *Factor_p1* and *Factor_p2* are calculated dependent on PRE/TRE state, according to the equations shown in Supplementary Figure 1.

Modify promoter

12. The value of *p1co* and *p2co* are calculated: *p1co* = *p1* x *Factor_p1*; *p2*co = *p2* x *Factor_p2*.
13. A site s1 is chosen at random.
14. If site s1 is F, it is changed to B with probability equal to *p1co*.
15. If site s1 is B, it is changed to F with probability equal to *p2co*.
16. The promoter state is evaluated by counting the number of sites in F and B configurations.
17. The values of *Factor_p3* and *Factor_p4* are calculated dependent on promoter state, according to the equations shown in Supplementary Figure 1.

DNA replication and mitosis

After the appropriate number of repetitions of steps 1-17 for a given cell cycle length:

18. Each nucleosome in the PRE/TRE is set to U with probability of 0.5.
19. All sites in the promoter are set to F.

4) Specific cases: Fitting to data

4.1) Memory of silencing

The model was fitted to experimental data documenting epigenetic memory of silencing of a transgenic reporter gene mediated by the *bxd* PRE/TRE during embryogenesis [4] (see Figure 2). The values of *p1* (promoter activation) were set to recapitulate the experimentally observed regulation of the reporter gene in the absence of the PRE/TRE, while *p2* (unbinding) was kept constant at 0.1 (Figure 2B, left panels and Supplementary Table 2A). The model promoter was silenced during cycles 1-13 (*p1* = 0.001), to reflect the absence of zygotic transcription during this stage [31]. The model promoter was activated in the posterior compartment at the onset of the initiation phase (2h:10, *p1* = 0.6), and subsequently activated also in the anterior compartment (4h:50, *p1*= 0.6) to simulate the loss of repressors in the anterior compartment [4]. The promoter parameters were selected to give the experimentally observed patterns and timing of transgenic reporter gene activity and to approximately reflect the known binding and dissociation rates of transcription factors in the range of seconds ([32], Supplementary Table 2A). The promoter was then coupled to the PRE/TRE, and parameters C_e, $C_{i,m}$, (*p3,p4*) and *p5* were fitted to obtain the range of values that best maintained memory of the promoter pattern established by the end of the initiation phase (4h:50) until 7.5 hours of embryogenesis (Supplementary Figure 2, Supplementary Table 1,

Supplementary Table 2A). Parameter sets that gave memory up to 7.5 hours also maintained this pattern in simulations until the end of adult development (data not shown).

In addition to the cell cycle lengths described above, the following constraints were imposed: The coupling strength between PRE/TRE and promoter was kept equal in both directions, and was kept constant within a given time window. Coupling during early cycles (C_e), was set to 0, since varying this parameter led to suboptimal memory (Figure 2F). Coupling during initiation and maintenance phases ($C_{i,m}$), was varied. The value of *p5* was kept constant throughout the simulation. The input values of *p3* and *p4* were equal to each other, and were kept equal to each other throughout the simulation. The values of (*p3,p4*) were adjusted during the simulation only by response to the promoter (Figure 1C) and not by any other adjustment. Thus the fitting shown in Supplementary Figure 2D was performed with three free parameters: $C_{i,m}$, (*p3, p4)*, and *p5*. The model was also constrained by a requirement for a uniform memory of silencing in the anterior compartment and of activation in the posterior, without variegation. These constraints allowed upper and lower limits for the feedback - independent (*p5*) and feedback – dependent parameters (*p3*, *p4*), and lower limits for the coupling parameter ($C_{i,m}$), to be determined (Supplementary Figure 2, Supplementary Table 2A).

To visualise the output of the model and compare it to reporter gene activity measured experimentally, model promoter states were averaged over time windows of 10 minutes, with the reasoning that experimentally detected reporter gene products (RNA or protein) would be measured as accumulated products at any given time point. The time window of 10 minutes was selected to give visual insights into the evolution of the system over time and does not affect the simulation itself, for which data are stored at every iteration.

4.2) Memory of activation

The model was fitted to experimental data documenting epigenetic memory of activation of a transgenic reporter gene mediated by the *Fab7* PRE/TRE following artificial activation by a one hour heat shock during embryogenesis and monitored as adult eye colour [5] (see Figure 3 and legend). We first established the model promoter activation during embryogenesis in the absence of the PRE/TRE. To this end, all coupling parameters were set 0, the parameter *p2* was kept constant at 0.1, and the values of *p1* were set to simulate the experimental situation in which no heat shock, later or early heat shock was given for one hour (Figure 3C and Supplementary Table

2A). The model promoter was silenced during cycles 1-13 (*p1* = 0.001), to reflect the absence of zygotic transcription during this stage [31]. The model promoter was then activated to basal levels at the onset of the initiation phase (2h:10, *p1* = 0.06, Figure 3C, Supplementary Table 2A). This basal promoter level was chosen to recapitulate the intermediate level of *mw* reporter gene activity in the absence of a PRE/TRE[5]. A heat shock was simulated by further activating the promoter for one hour, either at the onset of the maintenance phase (4h:50-5h:50, *p1* = 0.6, Figure 3C late), or at the onset of the initiation phase (2h:10-3h:10, *p1* = 0.6, Figure 3C early). After the heat shock, the promoter was returned to the basal level of activation (*p1* = 0.06) and the simulation was run until the end of adult development (216h or 9 days, Supplementary Table 1).

We then coupled this model promoter to the PRE/TRE, and performed parameter scans (Supplementary Figure 3). We imposed the same constraints on parameter values as described above for memory of silencing, and searched for values of (*p3*,*p4)*, *p5* and $C_{i,m}$ that enabled memory of promoter activation (Supplementary Figure 3). Remarkably, the same PRE/TRE parameter set that gave optimum memory of silencing in the previous experiment was able to recapitulate the observed memory of activation in the heat shock experiment (Figure 3, Supplementary Table 2A). Exploration of parameter space allowed upper and lower limits for the parameters (*p3*,*p4)* and *p5* and lower limits for $C_{i,m}$ to be determined (Supplementary Figure 3).

To visualise the output of the model during embryogenesis, promoter states were averaged over time windows of 10 minutes as described above for the silencing experiment. For the eye discs shown in Figure 3C-E, promoter states were averaged over 10 hour time windows, selected to represent the length of each cycle in the disc. The time windows were selected to give visual insights into the evolution of the system over time and do not affect the simulation itself, for which data are stored at every iteration. The time courses are shown in Figure 3 up to 100h (the end of the second mitotic wave in the 3rd instar larval eye disc, see Supplementary Table 1). After this time, no further cell division takes place and the model showed no average change in PRE/TRE or promoter status.

4.3) Modelling the third instar larval eye disc and fitting to the eya gradient

4.3.1) Model features: Conversion of temporal *eya* regulation into spatial eye disc gradient

Both the endogenous *eya* gene and the transgenic *eya1::GFP* reporter gene are activated in the 3rd instar larval eye disc in the zone of non-proliferating cells immediately anterior to the

morphogenetic furrow (MF) (Figure 6A,B zone 2) [33]. The furrow moves across the disc from posterior to anterior, leaving differentiating cells in its wake. Behind the furrow, *eya* expression decays spatially in a gradient pattern (Figure 6A,B, zone 3). Several of the transcription factors that activate *eya* remain present in zones 2 and 3 of the disc, and the repressors are continuously present in zone 1 [34,35]. Thus the *eya* gene is fundamentally different in its regulation to the *Hox* genes: it switches in specific cells from a silent to an active state late in development, it does not appear to require a classical memory of activation and repression (because the activators and repressors remain present), and it displays a gradient rather than an all-or-none pattern. The shape of the *eya* gradient both ahead of and behind the furrow depends on the PRE/TRE (Figure 6D-F).

In early 3rd instar, the MF and the zone of *eya* activation are initiated at the posterior of the disc, and move across the disc from posterior to anterior at the rate of approximately 3-4 cell rows per 70 mins [21]. Prior to the passage of the MF, cells arrest in G1 for 6-8 hours, and undergo a single coordinated mitosis (second mitotic wave) immediately afterwards [21]. After this, cells no longer proliferate. The entire duration of 3rd instar is approximately 48 hours (Supplementary Figure 8A).

To model *eya1::GFP* activation in the 3rd instar larval eye disc we performed different time course simulations, each representing a cell at a different position along the anterior - posterior axis of the disc (Supplementary Figure 8A). The eye disc is approximately 120 cells wide, from anterior to posterior [36,37]. These cells were modelled as 120 separate simulations. In each simulation, all cell cycles from cycle 1 of embryogenesis were included, and the simulation was run for a total of 120 hours (to the end of the third larval instar). Initial conditions: at the start of embryogenesis, all promoter sites were set to F and all PRE/TRE sites were set to U. Cells at each position in the disc along the anterior - posterior axis are exposed to the morphogenetic furrow at different time points. In the model, the time difference of exposure to the MF between adjacent cells is 24 minutes, i.e., the MF moves across the disc at an average rate of one cell row per 24 minutes, consistent with experimental observations [21]. For each position in the eye disc, the G1 arrest and *eya1::GFP* activation was set at an appropriate time (Supplementary Figure 8A), and the following steps were performed:

- G1 arrest and *eya1::GFP* activation: 8 hours
- One replication cycle
- *eya1::GFP* decay

In the absence of more quantitative time – resolved data we make two assumptions in the model: (1) we assume that the extent and speed of *eya* activation is the same in all cells of the disc (although it occurs at different time points, see Supplementary Figure 8A). (2) We assume that the MF progresses at a constant rate across the disc, although only average rates are available from literature [21].

For each simulated time course, the gradual activation and decay of *eya* over time was achieved by varying the value of *p1* and fitting to experimental data as described in section 4.3.2 below. In mid 3rd instar, (96 hours of development) the time point at which we performed our experiments and evaluated output of simulations, the MF and the zone of *eya* activation are approximately halfway across the disc (Figure 5, Supplementary Figure 8A). To generate a snapshot of the model disc at this time point, each simulated time course was averaged over a ten-minute window at 96h of development (or 24h into 3rd instar, see Supplementary Figure 8B). This was applied to each cell at each position of the disc, resulting in a row of 120 cells along the anterior - posterior axis. This operation was performed independently 20 times to generate 20 rows of cells. These 20 rows were compiled to generate the image shown in Supplementary Figure 8B (bottom), which represents a portion of the anterior-posterior axis of the disc (120 cells wide from anterior to posterior, and 20 cells deep from top to bottom). To generate an average profile of the promoter output across five discs, the promoter output at each of the 120 positions was averaged for 100 such rows (Supplementary Figure 8C). The single round of replication is visible as a dip in the profile between zones 2 and 3.

4.3.2) Fitting to experimental data: Fitting the promoter without PRE/TRE

Fitting the model to the experimental data was performed in two steps: first the promoter output without the PRE/TRE was fitted to data from *eya1::GFP* transgenes lacking the PRE/TRE, and subsequently the effect of the PRE/TRE on the promoter was fitted in the coupled system (Supplementary Figures 8G and 9A,B,E).

To fit the model to data without the PRE/TRE, the model was used without coupling, so that the model promoter output depended solely on the parameters *p1* and *p2*. *p2* (TF dissociation) was fixed at 0.1, and *p1* (TF association) was varied using Hill functions, to progressively and smoothly change the value of *p1* and to fit to the data (Figure 6D,F,L,N). The *p1* values for the segments of

the time course (before adjustment via Hill functions) are as follows: before *eya* activation (disc cycles 1-7), *p1disc* = 0.01; during *eya* activation (disc cycle 8), *p1eya1* = 0.6; during *eya* decay (after cycle 8), *p1eya2* = 0.25 (see also Supplementary Table 2A). Simulations were performed as follows:

- At each iteration (i) during eye disc cycle 8, (92h – 100h, see Supplementary Table 1) calculate UP_Hill = $K_a(i/t_a)^{\wedge} h_a/(1 + (i/t_a))^{\wedge} h_a)$, with Hill coefficient h_a = 3, constant K_a = 0.4, and time factor t_a = 600. Iteration i starts from 1 at the beginning of disc cycle 8. Each iteration is 12 sec.

- At step 12 (see core model above), the value of the parameter *p1co* is calculated as *p1co* = *p1eya1* x *Factor_p1* x UP_Hill (in simulations without coupling, *Factor_p1* = 1).

- After the last iteration of cycle 8, one replication is performed

- At each iteration (*i*) in post mitotic disc, (100h – 120h, see Supplementary Table 1) calculate DOWN_Hill = $K_d(i/t_d))^{\wedge} h_d /(1 + (i/t_d))^{\wedge} h_d)$, with Hill coefficient h_d = 2, constant K_d = 0.85, and time factor t_d = 100. Iteration *i* starts from 1 at the beginning of post mitotic disc. Each iteration is 12 sec.

- At step 12 (see core model above), the value of the parameter *p1co* is calculated as *p1co* = *p1eya2* x *Factor_p1* x DOWN_Hill (in simulations without coupling, *Factor_p1 = 1*).

The average profiles generated by the model (Figure 6N, Supplementary Figure 8C) and the experiment (Figure 6F, Supplementary Figure 8F) were compared (Supplementary Figure 8G). To this end, the experimental data and the model output were each discretized into 40 bins. The goodness of fit was evaluated as the mean squared difference between the data and the model for each of the 40 bins (see Supplementary Figure 9).

The shape of the gradient that we measure experimentally is affected by cell size and tissue shape: the cells in the MF are folded out of the plane of the disc epithelium, resulting in effective compression of the tissue around this point. These effects were included in the model by

appropriate conversion of the 120 simulation data points into the 40 model bins. We further assumed a constant rate of MF movement and *eya* activation as described above. In the absence of quantitative data these assumptions are well - justified. However, we found that because of these necessary simplifications, it was not possible to fit the model exactly to the experimental data (see Figure 6F,N). Thus we aimed to recapitulate the most important features of the profile in the absence of the PRE/TRE. These are:

- The complete repression of *eya*::*GFP* in zone 1. This was achieved in the model by setting *p1disc* to 0.01 in the repressed zone (See Supplementary Table 2A).
- The activation of *eya::GFP* in a smooth and steep manner within zone 2. This was achieved by varying *p1eya1* in time course simulations according to a Hill function (see above, and Supplementary Table 2A).
- The sharp drop in *eya::GFP* levels immediately following the second mitotic wave. This was achieved by including a replication step in the simulation, and resetting *p1eya1* to *p1eya2* immediately thereafter (See Supplementary Table 2A).
- The gradual decay in *eya::GFP* levels from anterior to posterior in zone 3. This was achieved by varying *p1eya2* according to a second Hill function (see above, see also Figure 6 and Supplementary Figure 8).

The timing of the cell cycle and postmitotic phase, the rate of passage of the MF and the total developmental time for third instar, in combination with the spatial criteria described above, were sufficient to place stringent constraints on the values of *p1* and the parameters for the Hill functions. For parameter values see Supplementary Table 2A.

4.3.2) Fitting to experimental data: Fitting the promoter with the PRE/TRE

To compare experimental data for the *eya::GFP* gradient with and without the PRE/TRE, profiles from eye discs of larvae carrying the two different transgenes were compared (Figure 6F). To this end, the average profiles from each genotype were aligned to each other using the MF as a reference point, and further scaled using the anterior edge of zone 2 and the posterior edge of the disc. The experimental data with the PRE/TRE were then discretized into 40 bins as described above. To fit the model to the data, the promoter parameters determined above from fitting without the PRE/TRE were fixed, coupling was introduced, and the PRE/TRE parameters (C_{eya}, (*p3, p4*),

and *p5*) were varied (Supplementary Figure 9E). C_e and $C_{i,m}$ (coupling prior to *eya* activation) were set to 0, as coupling during these early stages was found to prevent proper gradient formation (data not shown). Simulations were performed as described in section 4.3.2, with coupling. The model was compared to the experimental data with the PRE/TRE as described above, and parameter scans were performed (see Supplementary Figure 9E). Fitting was performed using experimental data from both the *eya* PRE/TRE (Figure 6F) and the *bxd* PRE/TRE (Figure 6H) enabling parameter values to be determined for both. This allowed optimal values for all four parameters ((*p3, p4*), *p5*, $C_{i,m}$ and C_{eya}) to be determined (Supplementary Figure 9).

For the *eya* PRE/TRE, the fitting was performed as described above, to optimise the fit to a single feature, namely the shape of the experimentally observed gradient, which showed a smooth pattern across the disc (Figure 6F, Supplementary Figure 9A,B,E). In contrast, for the *bxd* PRE/TRE, the experimental data showed two features: a specific average gradient shape (which is different from that produced by the *eya* PRE/TRE), and strong variegation (Figure 6G,H, Supplementary Figure 9C,D). To identify parameter space that fulfils both of these criteria, we fitted the gradient shape as for the *eya* PRE/TRE (Supplementary Figure 9F), and evaluated the extent of variegation separately (Supplementary Figure 9G). This was done as follows: For each parameter combination, 100 independent simulations were performed and profiles were generated. The standard deviation (SD) from the mean of these 100 simulations was scored at each position along the profile. SD was then averaged for the whole profile, giving a single value for each parameter combination. A high SD is caused by variegation (i.e. a mixture of active and silent states, as shown in Supplementary Figure 9C). A lower SD indicates that the promoter output is more similar from one simulation to the next (e.g., as in Supplementary Figure 9A). This identified a clear set of parameters that cause variegation (Supplementary Figure 9G) and that fit the gradient of the *bxd* PRE/TRE (Supplementary Figure 9F). The best fit was determined as the zone of overlap between these two parameter sets (the upper left corner in plots in Supplementary Figure 9F and G, for C_{eya} = 2.5 and 1.25 for models 1 and 2 respectively).

4.3.3) Fitting to experimental data: Exploring a model with two PRE/TREs

If two PRE/TREs instead of one are present in the *eya1::GFP* transgene (Figure 5A), then the ΔPRE/TRE construct would still contain a PRE/TRE at the transcription start site (TSS), and the observed gradient pattern would arise from an interaction between the enhancer and this PRE/TRE. Since we were not able to perform experiments to disrupt the putative promoter

PRE/TRE without also disrupting the TSS, we explored this scenario with modelling, using model 1 as a starting point. We made several simplifying assumptions in the model:

- The two *eya* PRE/TREs have identical size and properties.
- The *bxd* PRE/TRE has identical size to one of the *eya* PRE/TREs, but can have different properties (in terms of (*p3,p4)* and *p5*).
- Each PRE/TRE is identically coupled to the promoter, so that two PRE/TREs give two-fold higher coupling strength than a single one.
- When two PRE/TREs are present they are in communication with each other, such that recruitments at one PRE/TRE can modify a nucleosome in the other, with equal probability regardless of their linear distance from each other. The intronic *eya* PRE/TRE and the putative TSS PRE/TRE are 1 kb apart, and the whole region is covered with H3K27me3, so this is a reasonable assumption (Figure 5A).

More complex descriptions in which the two PRE/TREs have different properties and are differently coupled to the promoter are possible and can easily be modelled using this framework. Likewise, distance constraints could be added. However since we do not have data on the contributions of the putative promoter PRE/TRE to the output of the promoter, we chose to work with the simplest possible model. The alternative one-PRE/TRE and two-PRE/TRE models are shown in Supplementary Figure 10A-D.

We modelled the two-PRE/TRE scenario by increasing the total system size for the PRE/TRE to m=80 instead of m=40 nucleosomes. We then performed parameter scans by varying the values of (*p3,p4)* (feedback) and *p5* (feedback independent transitions) and fitting to experimental data. We also varied the coupling strength of each PRE/TRE to the promoter, but with the constraint that the coupling strength was two-fold higher for two PRE/TREs than for a single PRE/TRE.

We first searched for parameter combinations that gave the best fit to both the experimentally measured *eya1::GFP* gradient (modelled with two identical PRE/TREs) and to the experimentally measured *eya1::GFP ΔPRE/TRE* gradient (modelled with a single PRE/TRE, identical to one of these two). This analysis is shown in Supplementary Figure 10F, and compared to the equivalent parameter scans for the simpler single PRE/TRE model used in the main paper (Supplementary

Figure 10E). Interestingly the best fit of the two-PRE/TRE model to both data sets was obtained within a range of parameters that are similar to but more limited than those already determined for the single PRE/TRE model (compare panels E and F in Supplementary Figure 10). The best fit was obtained at a coupling strength of C_{eya} = 2.5 (Supplementary Figure 10F), of which half is contributed by each PRE/TRE in the two PRE/TRE model. Examples of the output of the parameter set that gave the best fit to the experimental data for both the two PRE/TRE model and the one PRE/TRE model are shown in Supplementary Figure 10 J,K,N,O, R, S ((*p3,p4)* = 0.2; *p5* = 0.17). We did not find parameters for the two-PRE/TRE model that gave a strong activation of the reporter ahead of the furrow (compare panels R and S in Supplementary Figure 10). However, the main features of the data, namely the sharpened gradient observed in the *eya1::GFP* data and the repression in the posterior part of the disc, are captured by both models. In summary, the modelling indicates that whether one or two PRE/TREs are present in the *eya* locus, these PRE/TREs need to allow dynamic change in promoter output. In the model this is achieved by a relatively high value of feedback- independent transitions (*p5* = 0.17) which enables the PRE/TRE(s) to respond flexibly to the promoter.

We next examined the effect on the model output of swapping one PRE/TRE with a different PRE/TRE. To this end, we fixed the properties of one PRE/TRE, representing the *eya* TSS PRE/TRE, by setting the value of (*p3,p4)* to 0.2 and of *p5* to 0.17. We then systematically varied the (*p3,p4)* and *p5* values for the other PRE/TRE, representing the intronic PRE/TRE in the *eya1::GFP* transgene, which was replaced in the experiment with the *bxd* PRE/TRE. We varied C_{eya} for both PRE/TREs. We performed parameter scans to determine values of (*p3,p4)* and *p5* for the second PRE/TRE, and values of C_{eya} for the entire system as above, that gave the best fit to the experimental data observed for the *bxd* PRE/TRE in the context of the *eya1::GFP* transgene (Supplementary Figure 10H). In addition we evaluated variegation as a required condition for fitting *bxd* data as described in section 4.3.2 above (Supplementary Figure 10I). This analysis identified a range of the parameters ((*p3,p4)*, *p5* and C_{eya}) that gave the best fit to the data. These parameter values were similar but more limited in range compared to those defined for the one-or-no PRE/TRE model (compare panels G and H in Supplementary Figure 10).

Examples of the output of the parameter set that gave the best fit to the experimental data for both the two PRE/TRE model and the one PRE/TRE model are shown in Supplementary Figure 10 L,M,P,Q,T,U ((*p3,p4)* = 0.25; *p5* = 0.04, C_{eya} for two PRE/TREs = 2.5). Both models show

variegation in the presence of the *bxd* PRE/TRE. The two-PRE/TRE model shows a more hybrid behaviour, meaning that the variegation is less black and white than for the one-PRE/TRE model (compare Supplementary Figure 10 panels P and Q). However, the main features of the data, namely the variegating gradient of reporter expression with higher expression levels anterior to the furrow, are captured by both models. In summary, this analysis indicates that if two PRE/TREs are present in the *eya* locus, the replacement of one of these with a PRE/TRE that has identical properties to those determined for the *bxd* PRE/TRE in the one-PRE/TRE model is sufficient to cause the system to variegate, whilst still responding to the gradient pattern of the promoter. In the model, the important difference between the *eya* and *bxd* PRE/TREs is the value of the parameter *p5*, which needs to be low for *bxd* (*p5* = 0.04) to cause the model system to variegate, and high for *eya* (*p5* = 0.17), to allow the model system to respond flexibly to the promoter.

Since both models are able to recapitulate the main features of the data, and both can be fitted with similar parameter sets, we chose to use the simplest model (one PRE/TRE) for the main experiments shown in the paper.

Supplementary discussion

Here we discuss specific issues that were not included in the main manuscript due to space constraints.

Early cycles and transgenerational inheritance

We show that the early rapid division cycles are essential for keeping the system in a naïve state prior to the initiation phase. Many organisms begin life with relatively rapid cell cycles, which become slower later in development [38-40]. Thus the concept of cell cycle length as a regulator of epigenetic stability is globally relevant. Interestingly, fly and vertebrate PcG and TrxG proteins regulate several genes that control the cell cycle [41-44], indicating that the interplay between cell cycle length and PcG/TrxG regulation may be tightly regulated.

Early naïve chromatin states, generated by germline reprogramming, are essential for correct development [45]. We propose here that in addition to the events in the germline, effective reprogramming in *Drosophila* may also require the early rapid zygotic divisions. Indeed our model predicts that chromatin marks and bound proteins do not survive the constant dilution of parental

histones that occurs during cycles 1-13. However, several authors have reported intergenerational and transgenerational inheritance of chromatin or gene expression states for specific transgenes or loci in *Drosophila* [5,46-48]. These observations suggest that specific loci (with specific PRE/TREs) may be able to resist reprogramming due to their local properties, whilst the rest of the genome is reprogrammed. Alternatively, intergenerational inheritance may be mediated by chromatin – independent mechanisms.

The maternal to zygotic transition at cycle 14

We show in the model that the exit from rapid cycling that occurs at cycle 14 is decisive for enabling the model system to recover from the constant disruptions of replication and to begin to stabilise. Many chromatin changes have been observed to occur at or after cycle 14 in the *Drosophila* embryo. This time point marks the transition from maternal to zygotic transcription [31,49,50]**.** Chromatin changes that correspond to this time point include the accumulation of specific histone modifications at enhancers [1], increased residence time of PcG proteins on chromatin [51,52], the emergence of long-range chromatin interactions [53] and somatic pairing of homologous chromosomes [54]. Interestingly, several of these features have been shown to be independent of transcription [53,54], suggesting that they are not simply caused by the increase in zygotic transcription that occurs at this time point. We propose that these changes may in part be due to the exit from rapid cycling that occurs at cycle 14, allowing chromatin conformations and components to accumulate without the constant disruptions of replication and mitosis that occur during cycles 1 - 13. Our prediction and observation of early accumulation of histone marks in pole cells, which exit the cell cycle at cycle 10, is consistent with this idea (Figure 4).

Coupling strength and mechanism

We have shown theoretically that regulated coupling is essential for the model to recapitulate different experimental results. The strength of coupling required in the model to fit experimental observations depends on the experiment in question. Stronger coupling was required for accurate memory of silencing and activation (C = 4, model 1) than to model the dynamic *eya* gradient (C = 2.5, model 1). A value of C = 4 corresponds to a 55 - fold bias of the PRE/TRE or promoter towards one or other extreme state in response to the corresponding extreme state of the other element. For the *eya* gradient, C = 2.5 for model 1 corresponds to a 12 - fold bias. Although regulated coupling is essential in the model, its molecular meaning in biology is not clear, thus it is difficult to assess whether the coupling strengths given above are feasible in molecular terms. We note

however, that a PRE/TRE can give up to 48 – fold repression of a linked reporter gene [22], thus large coupling effects do exist, although their molecular mechanisms are unclear.

The molecular mechanisms of coupling between PRE/TREs and promoters is unknown. One of the most compelling potential coupling mechanisms is *trans*-regulation by homologous pairing. *Drosophila* PRE/TREs silence a reporter more strongly when homozygous than when heterozygous. This phenomenon, known as pairing sensitive silencing (PSS) is thought to involve physical contacts between the homologs[55]. PSS has been shown to vary quantitatively between transgenic loci and for different PRE/TREs[13]. Physical pairing of homologous alleles of the BX-C is first detected in embryos during nuclear division 13, and reaches maximum levels after gastrulation (cycle 14, stage 7, ca. 3h) [54]. Thus PSS is a good candidate for a coupling mechanism, because it increases the effect of the PRE/TRE on the promoter, and it is locus – specific and developmentally regulated, coming into play during cycle 14. Interestingly, pairing sensitive activation (PSA) has also been observed for some PRE/TRE reporters [56]. Thus pairing – dependent mechanisms may be equally relevant to silencing and activation.

Supplementary References

1 Li, X. Y., Harrison, M. M., Villalta, J. E., Kaplan, T. & Eisen, M. B., Establishment of regions of genomic activity during the Drosophila maternal to zygotic transition. eLife 3 (2014).

2 Bonnet, J. *et al.*, Quantification of Proteins and Histone Marks in Drosophila Embryos Reveals Stoichiometric Relationships Impacting Chromatin Regulation. Dev Cell 51, 632-644 e636 (2019).

3 Steffen, P. A. & Ringrose, L., What are memories made of? How Polycomb and Trithorax proteins mediate epigenetic memory. Nat Rev Mol Cell Biol 15, 340-356 (2014).

4 Chan, C. S., Rastelli, L. & Pirrotta, V., A Polycomb response element in the Ubx gene that determines an epigenetically inherited state of repression. EMBO J 13, 2553-2564. (1994).

5 Cavalli, G. & Paro, R., The Drosophila Fab-7 chromosomal element conveys epigenetic inheritance during mitosis and meiosis. Cell 93, 505-518 (1998).

6 Dodd, I. B., Micheelsen, M. A., Sneppen, K. & Thon, G., Theoretical analysis of epigenetic cell memory by nucleosome modification. Cell 129, 813-822 (2007).

7 Angel, A., Song, J., Dean, C. & Howard, M., A Polycomb-based switch underlying quantitative epigenetic memory. Nature 476, 105-108 (2011).

8 Haerter, J. O., Lovkvist, C., Dodd, I. B. & Sneppen, K., Collaboration between CpG sites is needed for stable somatic inheritance of DNA methylation states. Nucleic Acids Res 42, 2235-2244 (2014).

9 Ringrose, L., Noncoding RNAs in Polycomb and Trithorax Regulation: A Quantitative Perspective. Annu Rev Genet 51, 385-411 (2017).

10 Sneppen, K. & Ringrose, L., Theoretical analysis of Polycomb-Trithorax systems predicts that poised chromatin is bistable and not bivalent. Nature Communications in press (2019).

11 Berry, S., Dean, C. & Howard, M., Slow Chromatin Dynamics Allow Polycomb Target Genes to Filter Fluctuations in Transcription Factor Activity. Cell Syst 4, 445-457 e448 (2017).
12 Herzog, V. A. *et al.*, A strand-specific switch in noncoding transcription switches the function of a Polycomb/Trithorax response element. Nat Genet 46, 973-981 (2014).
13 Okulski, H., Druck, B., Bhalerao, S. & Ringrose, L., Quantitative analysis of polycomb response elements (PREs) at identical genomic locations distinguishes contributions of PRE sequence and genomic environment. Epigenetics Chromatin 4, 4 (2011).
14 Bauer, M., Trupke, J. & Ringrose, L., The quest for mammalian Polycomb response elements: are we there yet? Chromosoma 125, 471-496 (2016).
15 Comet, I. *et al.*, PRE-mediated bypass of two Su(Hw) insulators targets PcG proteins to a downstream promoter. Dev Cell 11, 117-124 (2006).
16 Yang, H. *et al.*, Distinct phases of Polycomb silencing to hold epigenetic memory of cold in Arabidopsis. Science 357, 1142-1145 (2017).
17 Foe, V. E. & Alberts, B. M., Studies of nuclear and cytoplasmic behaviour during the five mitotic cycles that precede gastrulation in Drosophila embryogenesis. J Cell Sci 61, 31-70 (1983).
18 Foe, V. E., Mitotic domains reveal early commitment of cells in Drosophila embryos. Development 107, 1-22 (1989).
19 Lawrence, P. A. *The Making of A Fly: The Genetics of Animal Design*. (Blackwell Scientific Publications, 1992).
20 Xin, S., Weng, L., Xu, J. & Du, W., The role of RBF in developmentally regulated cell proliferation in the eye disc and in Cyclin D/Cdk4 induced cellular growth. Development 129, 1345-1356 (2002).
21 Escudero, L. M. & Freeman, M., Mechanism of G1 arrest in the Drosophila eye imaginal disc. BMC developmental biology 7, 13 (2007).
22 Martínez-Balbás, M. A., Dey, A., Rabindran, S. K., Ozato, K. & Wu, C., Displacement of sequence-specific transcription factors from mitotic chromatin. Cell 83, 29-38 (1995).
23 Parsons, G. G. & Spencer, C. A., Mitotic repression of RNA polymerase II transcription is accompanied by release of transcription elongation complexes. Molecular and cellular biology 17, 5791-5802 (1997).
24 Spencer, C. A., Kruhlak, M. J., Jenkins, H. L., Sun, X. & Bazett-Jones, D. P., Mitotic transcription repression in vivo in the absence of nucleosomal chromatin condensation. The Journal of cell biology 150, 13-26 (2000).
25 Kadauke, S. & Blobel, G. A., Mitotic bookmarking by transcription factors. Epigenetics Chromatin 6, 6 (2013).
26 Radman-Livaja, M. *et al.*, Patterns and mechanisms of ancestral histone protein inheritance in budding yeast. PLoS Biol 9, e1001075 (2011).
27 Alabert, C. *et al.*, Two distinct modes for propagation of histone PTMs across the cell cycle. Genes Dev 29, 585-590 (2015).
28 Petruk, S. *et al.*, TrxG and PcG Proteins but Not Methylated Histones Remain Associated with DNA through Replication. Cell 150, 922-933 (2012).
29 Francis, N. J., Follmer, N. E., Simon, M. D., Aghia, G. & Butler, J. D., Polycomb proteins remain bound to chromatin and DNA during DNA replication in vitro. Cell 137, 110-122 (2009).
30 Leung, K. H., El Hassan, M. A. & Bremner, R., A rapid and efficient method to purify proteins at replication forks under native conditions. BioTechniques 55, 204-206 (2013).
31 Harrison, M. M. & Eisen, M. B., Transcriptional Activation of the Zygotic Genome in Drosophila. Curr Top Dev Biol 113, 85-112 (2015).

32 Mueller, F., Wach, P. & McNally, J. G., Evidence for a common mode of transcription factor interaction with chromatin as revealed by improved quantitative fluorescence recovery after photobleaching. Biophys J 94, 3323-3339 (2008).
33 Bui, Q. T., Zimmerman, J. E., Liu, H., Gray-Board, G. L. & Bonini, N. M., Functional analysis of an eye enhancer of the Drosophila eyes absent gene: differential regulation by eye specification genes. Dev Biol 221, 355-364 (2000).
34 Kumar, J. P., The molecular circuitry governing retinal determination. Biochim Biophys Acta 1789, 306-314 (2009).
35 Ringrose, L., Polycomb comes of age: genome-wide profiling of target sites. Curr Opin Cell Biol 19, 290-297 (2007).
36 Ready, D. F., Hanson, T. E. & Benzer, S., Development of the Drosophila retina, a neurocrystalline lattice. Dev Biol 53, 217-240 (1976).
37 Tsao, C. K., Ku, H. Y. & Sun, Y. H., Long-term Live Imaging of Drosophila Eye Disc. Journal of visualized experiments : JoVE (2017).
38 Paranjpe, S. S. & Veenstra, G. J., Establishing pluripotency in early development. Biochim Biophys Acta 1849, 626-636 (2015).
39 Prados, F. J., Debrock, S., Lemmen, J. G. & Agerholm, I., The cleavage stage embryo. Human reproduction 27 Suppl 1, i50-71 (2012).
40 Farrell, J. A. & O'Farrell, P. H., From egg to gastrula: how the cell cycle is remodeled during the Drosophila mid-blastula transition. Annu Rev Genet 48, 269-294 (2014).
41 Oktaba, K. *et al.*, Dynamic regulation by polycomb group protein complexes controls pattern formation and the cell cycle in Drosophila. Dev Cell 15, 877-889 (2008).
42 Martinez, A. M., Colomb, S., Dejardin, J., Bantignies, F. & Cavalli, G., Polycomb group-dependent Cyclin A repression in Drosophila. Genes Dev 20, 501-513 (2006).
43 Gil, J., Bernard, D., Martinez, D. & Beach, D., Polycomb CBX7 has a unifying role in cellular lifespan. Nat Cell Biol 6, 67-72 (2004).
44 Liu, H., Cheng, E. H. & Hsieh, J. J., Bimodal degradation of MLL by SCFSkp2 and APCCdc20 assures cell cycle execution: a critical regulatory circuit lost in leukemogenic MLL fusions. Genes Dev 21, 2385-2398 (2007).
45 Iovino, N., Drosophila epigenome reorganization during oocyte differentiation and early embryogenesis. Briefings in functional genomics 13, 246-253 (2014).
46 Cavalli, G. & Paro, R., Epigenetic inheritance of active chromatin after removal of the main transactivator. Science 286, 955-958 (1999).
47 Ost, A. *et al.*, Paternal diet defines offspring chromatin state and intergenerational obesity. Cell 159, 1352-1364 (2014).
48 Zenk, F. *et al.*, Germ line-inherited H3K27me3 restricts enhancer function during maternal-to-zygotic transition. Science 357, 212-216 (2017).
49 Blythe, S. A. & Wieschaus, E. F., Coordinating Cell Cycle Remodeling with Transcriptional Activation at the Drosophila MBT. Curr Top Dev Biol 113, 113-148 (2015).
50 Perino, M. & Veenstra, G. J., Chromatin Control of Developmental Dynamics and Plasticity. Dev Cell 38, 610-620 (2016).
51 Ficz, G., Heintzmann, R. & Arndt-Jovin, D. J., Polycomb group protein complexes exchange rapidly in living Drosophila. Development 132, 3963-3976 (2005).
52 Fonseca, J. P. *et al.*, In vivo Polycomb kinetics and mitotic chromatin binding distinguish stem cells from differentiated cells. Genes Dev 26, 857-871 (2012).
53 Hug, C. B., Grimaldi, A. G., Kruse, K. & Vaquerizas, J. M., Chromatin Architecture Emerges during Zygotic Genome Activation Independent of Transcription. Cell 169, 216-228 e219 (2017).

54 Gemkow, M. J., Verveer, P. J. & Arndt-Jovin, D. J., Homologous association of the Bithorax-Complex during embryogenesis: consequences for transvection in Drosophila melanogaster. Development 125, 4541-4552 (1998).
55 Kassis, J. A., Unusual properties of regulatory DNA from the Drosophila engrailed gene: three pairing-sensitive sites within a 1.6-kb region. Genetics 136, 1025-1038 (1994).
56 Dejardin, J. & Cavalli, G., Chromatin inheritance upon Zeste-mediated Brahma recruitment at a minimal cellular memory module. EMBO J 23, 857-868 (2004).
