# Supplementary material for: A theoretical model of Polycomb/Trithorax action unites stable epigenetic memory and dynamic regulation
Source: Nat Commun. 2020 Sep 22;11:4782. doi: 10.1038/s41467-020-18507-4 (PMC7508846; doi:10.1038/s41467-020-18507-4)
Supplement: Supplementary file 3 — Description of Additional Supplementary Files [file 41467_2020_18507_MOESM3_ESM.pdf]

Description of Additional Supplementary Files

File Name: Supplementary Software

Description: The program was written in Matlab R2015b. The installation, run times and instructions for use are given in the document: Reinig_user_manual.pdf. The user can choose a plot option from 1 to 7 and a model from 1-14. The outputs correspond to the figures in the paper as outlined in the user manual. The code is available at https://github.com/Ringrose546/Beyond_memory_V2
