# Supplementary material for: A theoretical model of Polycomb/Trithorax action unites stable epigenetic memory and dynamic regulation
Source: Nat Commun. 2020 Sep 22;11:4782. doi: 10.1038/s41467-020-18507-4 (PMC7508846; doi:10.1038/s41467-020-18507-4)

User Manual for Simulation Program

`PRE_Promoter_Reinig.m`

Accompanying the manuscript:

"A theoretical model of Polycomb/Trithorax action unites stable epigenetic memory and dynamic regulation"

Leonie Ringrose

04.07.2020

Humboldt Universität zu Berlin, IRI- Lifesciences, Philippstr. 13, 10115 Berlin, Germany

leonie.ringrose@hu-berlin.de


```
% Copyright Leonie Ringrose, 04.07.2020

   %Licensed under the Apache License, Version 2.0 (the "License");
   %you may not use this file except in compliance with the License.
   %You may obtain a copy of the License at

   %http://www.apache.org/licenses/LICENSE-2.0

   %Unless required by applicable law or agreed to in writing, software
   %distributed under the License is distributed on an "AS IS" BASIS,
   %WITHOUT WARRANTIES OR CONDITIONS OF ANY KIND, either express or implied.
   %See the License for the specific language governing permissions and
   %limitations under the License.
```

1) General information

The program was written in Matlab R2015b. Runtimes are given for Mac Book Air (Processor 2,2 GHz Intel Core i7, Memory 8 GB 1600 MHz DDR3). The code is available at https://github.com/Ringrose546/Beyond_memory_V2

Install the following scripts in the same folder in Matlab:

PRE_Promoter_Reinig.m - *main script which calls all functions.*
plot_option_1_3.m
plot_option_2.m
plot_option_4.m
plot_option_5.m
plot_option_6.m
plot_option_7.m
lifetimefunction.m
memory_inputs.m
rep_function.m
statefunction.m
timecourse_function.m
timecourse_functionDOWNHILL.m
timecourse_functionUPHILL.m

ChIP_plots.m - *standalone script which creates the plots in Figure 4E of Reinig et al*

2) Plot options. In PRE_Promoter_Reinig.m, the user can choose a plot option from 1 to 7 and a model from 1-14 (see Figure S1 of Reinig et al). The outputs correspond to the figures in the paper as outlined in the following documentation.

```
% This script and the accompanying functions run all the simulations in Reinig et al.
% "A theoretical model of Polycomb/Trithorax action unites stable epigenetic memory and dynamic regulation"
% User defined plot_options 1-7 correspond to the figures as listed below.

% Plot options
%-------------------------------------------------------------------------------------

% 1: embryo: memory of silencing. Used for plots in Fig. 2 and S2 of the paper.
% 2: embryo: memory of silencing, parameter space analysis. Used for plots in Fig S1 and S2.
% 3: embryo: memory of activation, heat shock experiment. Used for plots in Fig. 3 and S3.
% 4: embryo: memory of activation, parameter space analysis. Used for plots in Fig S1 and S3.
% 5: eye disc: used for plots in Fig. 5 and S8.
% 6: eye disc: parameter space analysis. Used for plots in Fig. S1 and S8 of the paper.
    % N.B. this is a long simulation: n= 100 requires 1h approx!
% 7: general: time course for whole run and state analysis for the last 10 min window.
    % Used for histone level predictions(Fig. 4a)
```

3) User defined inputs. Other user-defined inputs are as follows: These are explained in more detail under each plot option.

```
% User-defined inputs. To run in default mode, just select a plot_option (1-7).
% Use defaults for all other inputs.
% This will run different simulations corresponding to the figures in the paper.
%-----------------------------------------------------------------------------------------------------------------------------
note = '20.06.20, Test';    % Use this space for any notes.
plot_option = 1;            % Choose 1-7. See above for list.
model = 1;                  % Define model: Choose 1 - 14. Default = 1. Model 2 gives similar results.
n = 50;                     % Number of total repeats of simulation. Default = 50. 100 for parameter scans (see user manual).

Coupling_Strength = 1;      % User defined coupling factor. Default = 1.
                            % NB the coupling factors given here will reproduce the figures in the User Manual. The revised
                            % version of the paper (June 2020) uses a coupling strength of 0.5 for Figures 2 and 3 only.
PRE_init = ('U');           % Define the initial state of the PRE/TRE (A, U or M). Default = U
Enhancer_init = ('F');      % Define the initial state of the promoter (B or F). Default = F

heatshock = 1;     % Plot option 3, 4 and 7. Choose 0 (none) 1 (early) or 2 (late). Ignored by other plot options.
PRE_ID = ('b');    % Plot option 5 and 7. PRE/TRE identity (e = eya or b = bxd). All other plot options use bxd parameters.

PcG = 1;           % Plot option 1, 3, 5, 7. Choose 1 (wild type) or 0 (mutant). Loss of PcG at onset of cycle 15
TrxG = 1;          % Plot option 1, 3, 5, 7. Choose 1 (wild type) or 0 (mutant). Loss of TrxG at onset of cycle 15

%End of user-defined section. Make changes beyond this point at your peril!
%-----------------------------------------------------------------------------------------------------------------------------
```

4) Outputs. The next pages describe plot options 1-7 with inputs as shown, and give examples of the plots that are generated by each. The parameters are automatically selected for each plot option and each model, according to Table S1 of the paper.

Plot option 1. Runs a time course of embryo development up to 7.5 hours, with and without coupling between the PRE/TRE and the promoter. Promoter is activated during development and the ability of the PRE/TRE to maintain silencing is evaluated.

Inputs:

```
note = 13.05.19, Model 1, test';
plot_option = 1;
model = 1;
n = 50;.

Coupling_Strength = 1;
PRE_init = ('U');
Enhancer_init = ('F');
```

Run time ca. 30 sec for n = 50

Outputs:

Figure 1. Time course of embryo with separate anterior and posterior compartments, as shown in figure 2B, C and S2A of the paper. Note that due to the stochastic nature of the model, different runs give slightly different results. Selecting model 2 for this plot option gives similar results. Other models do not perform as well as models 1 and 2 (see Figure S1 for description of models).

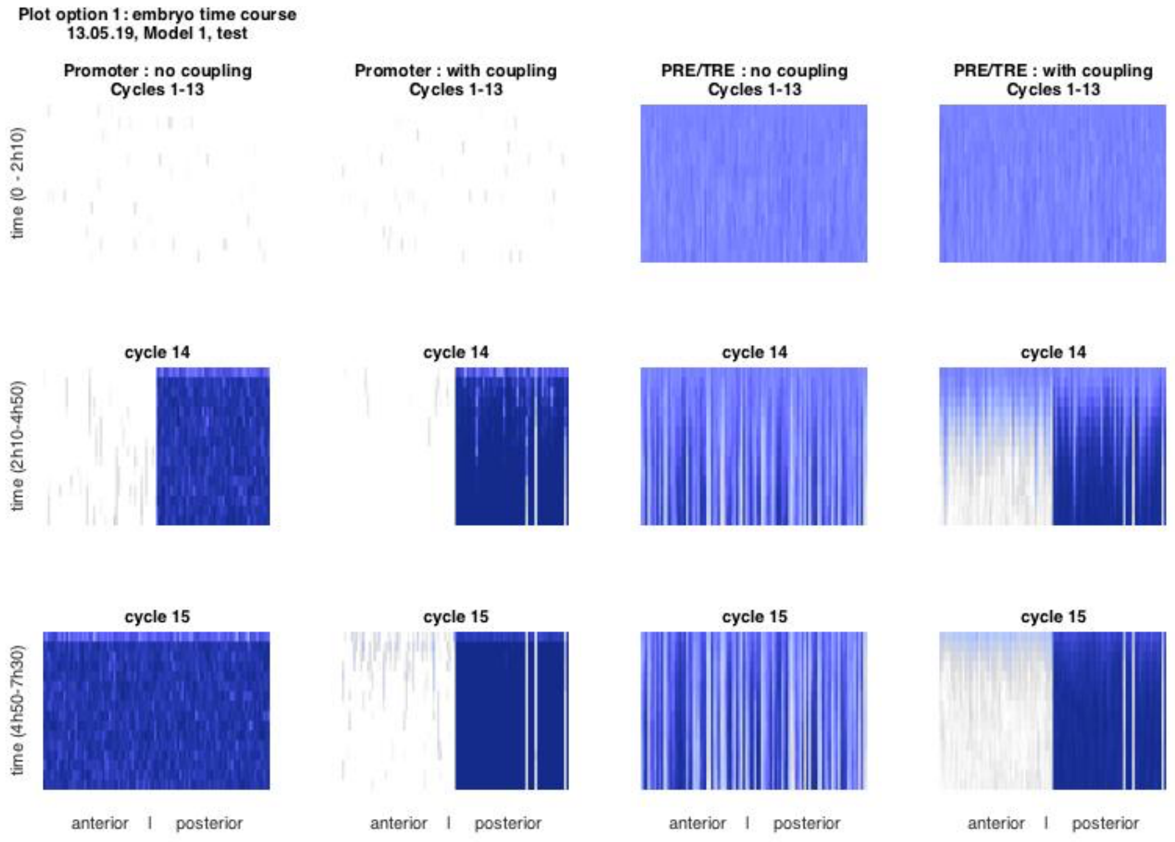

Figure 2. Average state of PRE/TRE and promoter for the last 10 minutes of the simulations shown above, as shown in Figure S2B of the paper. A (anterior) and P (posterior) compartments are shown separately. Top: Mean and standard deviation across the 50 independent simulations are shown. Bottom: the same data are plotted as box and whisker plots (Matlab default settings, see Figure 2 of Reinig et al for details). Note that the PRE/TRE shows high variability (variegation) unless coupled to the promoter.

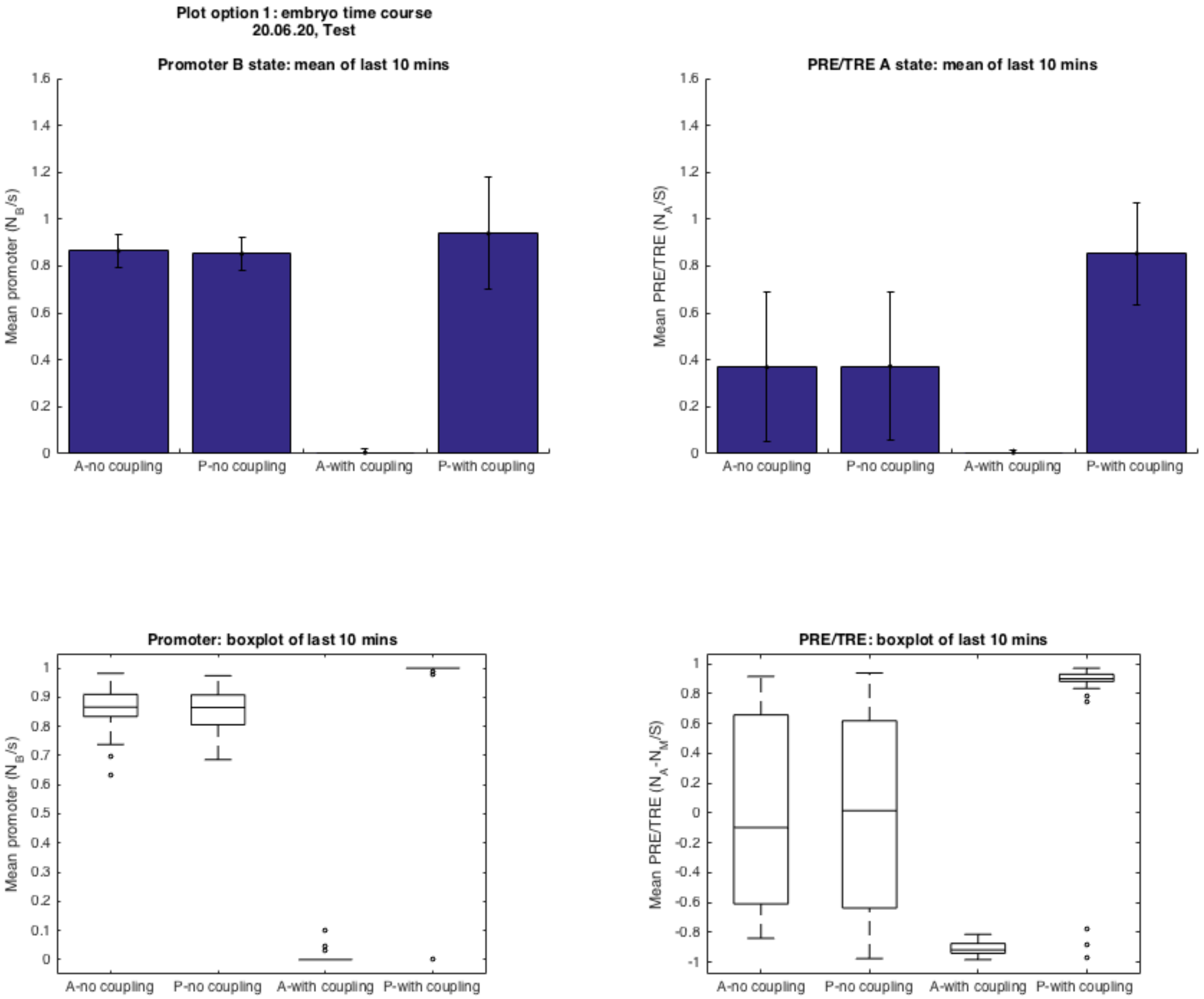

Figure 3. The memory score as shown in Figure 2 of the paper is calculated for the anterior compartment and plotted as mean and standard deviation (top) and as a box and whisker plot (bottom). A memory score of 1 indicates that the promoter state with the PRE/TRE at the end of the maintenance phase is close to that seen without the PRE/TRE at the end of the initiation phase (see Figure 2 of the paper for details, and Figure S2C for calculation of memory score.)

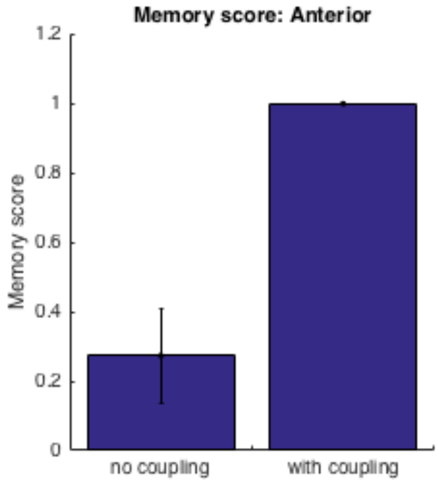

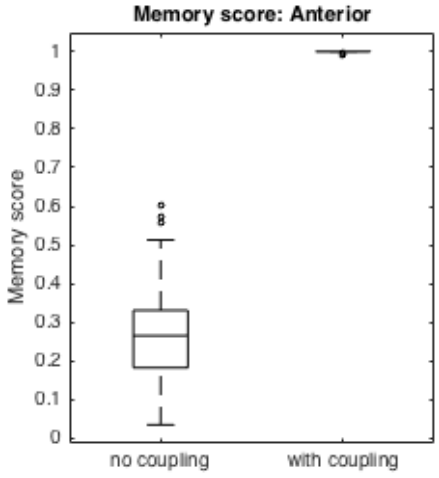

Plot option 2. Runs a parameter scan for the time course simulations shown in Plot option 1, and evaluates memory of initiated promoter states.

Inputs:

```
note = '13.05.19, Model 1, test';
plot_option = 2;
model = 1;
n = 100;

Coupling_Strength = 1;
PRE_init = ('U');
Enhancer_init = ('F');
```

Run time ca. 12 mins for n = 100

Input variation:

Model: Other models will give different results as shown in Figure S1G, H.
Coupling_Strength: See Figure S2D. The parameter scan is performed under a single coupling regime. A user- defined coupling strength of 1 defines the parameter $C_{i,m}$ for model 1 as 8, and for model 2 as 4, as shown in figure S2D. Other panels of figure S2D are generated by adjusting Coupling_Strength to 0, 0.25, or 0.5, and/or selecting model 2.

Outputs:

Figure 1. Parameter scan with evaluation of memory of silencing as explained in Figure S2C and D of the paper. The plot shows the promoter (left, as shown in Fig S2D) and the PRE/TRE (right, not shown in the paper). Yellow colour indicates good memory. See Fig. S2C for calculation of memory score.

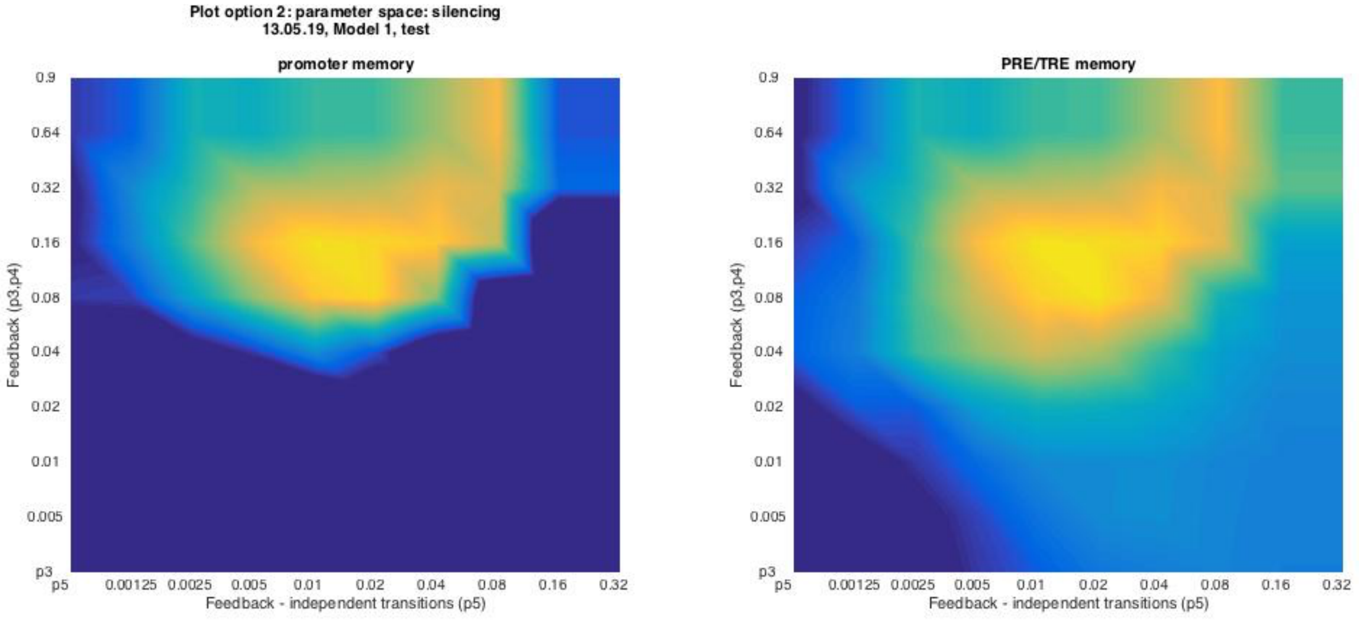

Figure 2. All raw data from parameter scan (not shown in paper). Each plot shows data averaged over last 10 minutes of the phase indicated. "Initiation": (4h40- 4h50)." Maintenance": (7h20 – 7h30). X-axis shows parameter values in blocks for a given value of parameter 2 (here, p5). Within each block, the values of parameter 1 were varied as indicated below the plots (here (p3,p4)). Pairs of columns represent anterior (left) and posterior (right) of the embryo for each parameter combination. Blue indicates active promoter or PRE/TRE. Vertical axis shows 100 repetitions for the same condition.

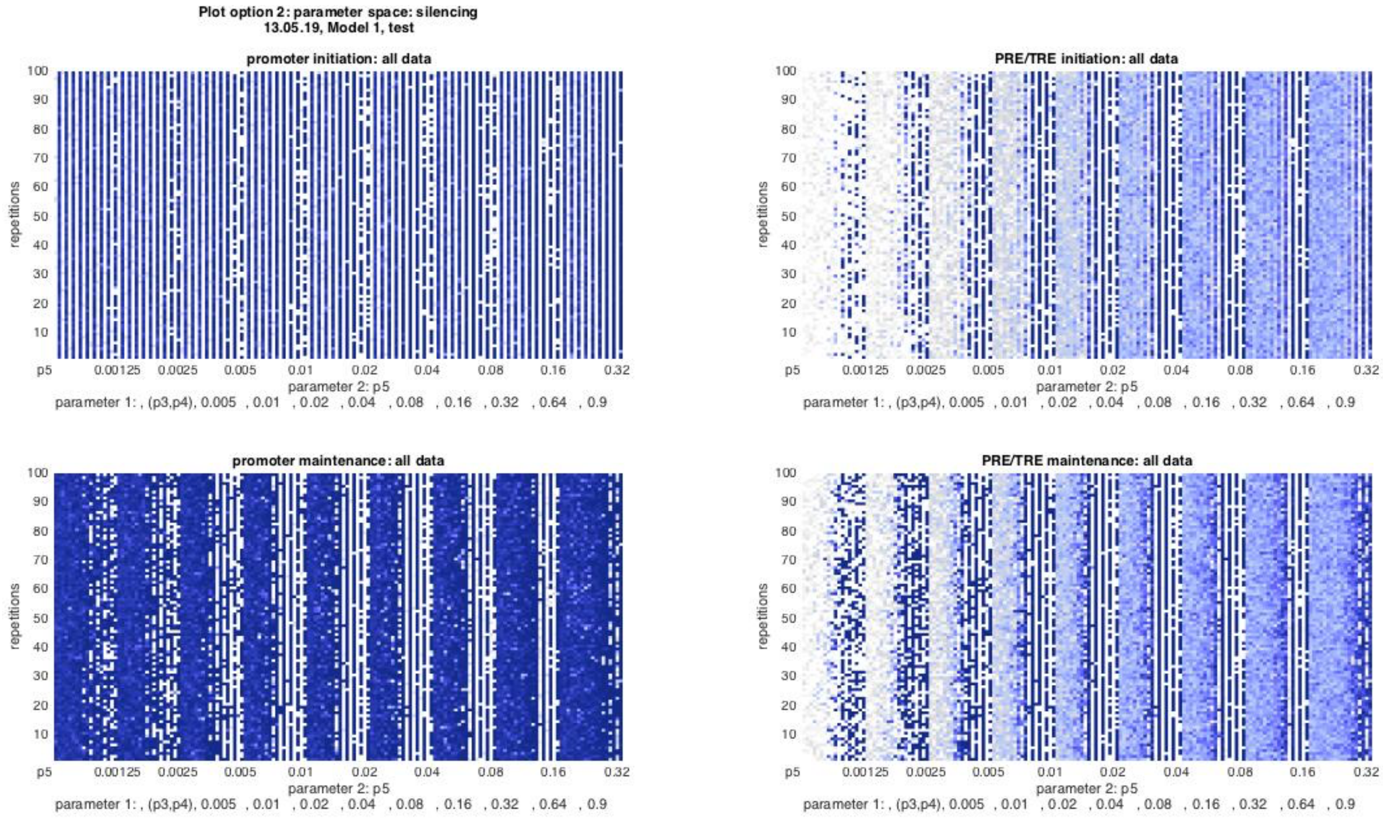

Figure 3. Data from parameter scan averaged over 100 repetitions showing mean and SD. Promoter levels in anterior and posterior for each parameter set are shown. Top: promoter memory, calculated as in Fig S2C. High "silencing memory" score indicates high maintenance of silencing in anterior and activation in posterior.

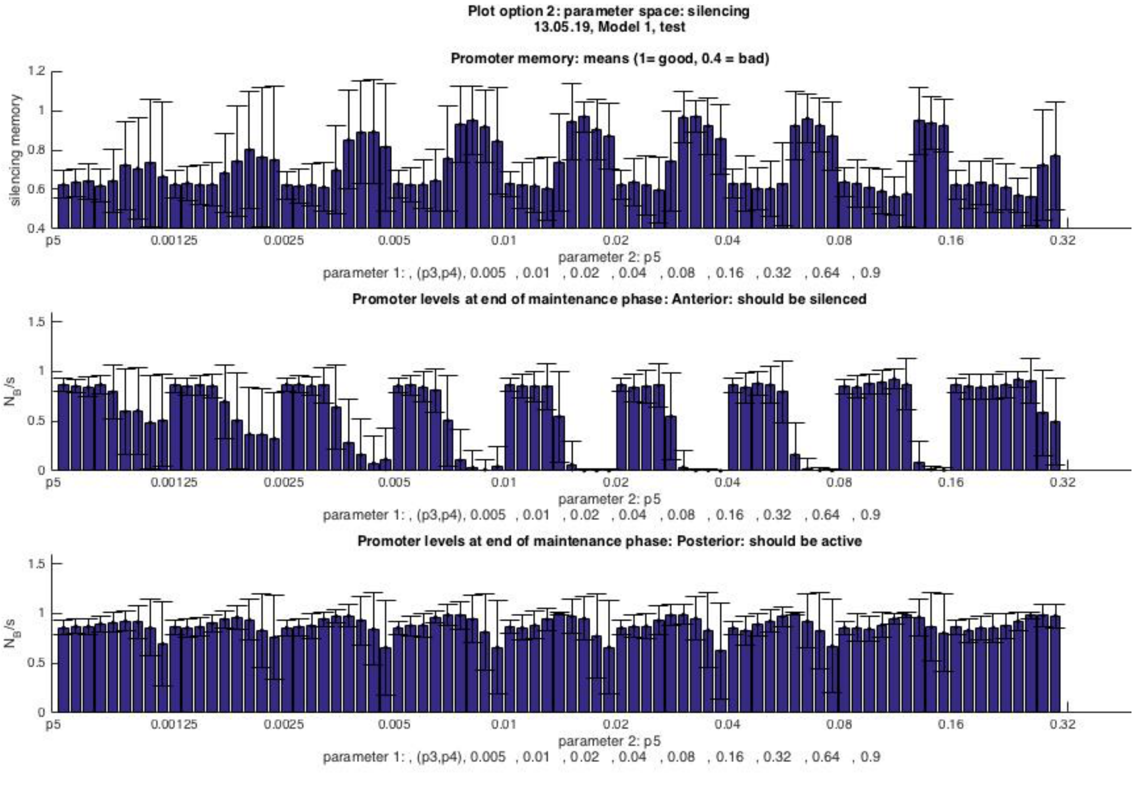

Plot option 3. Runs a time course of embryo development up to 7.5 hours, with and without coupling between the PRE/TRE and the promoter. Promoter is activated by the user at different time points during development and the ability of the PRE/TRE to maintain activation is evaluated.

Inputs:

```
note = '13.05.19, Model 1, test';
plot_option = 3;
model = 1;
n = 50;

Coupling_Strength = 1;
PRE_init = ('U');
Enhancer_init = ('F');

heatshock = 1;
```

Run time ca. 30 sec for n = 50

Input variation:

Heat shock: The user defines the heat shock timing by entering 0 (no heat shock) 1 (early heat shock, shown here) or 2 (late heat shock). Use model 1 to generate the data in Figure 3C-E, and model 1 or 2 for data in Figure S3A.

Outputs:

Figure 1. Time course of embryo, as shown in figure 3C-E and S3A of the paper. Note that anterior and posterior compartments are shown separately as for plot option 1, but the heatshock is applied identically in both. Note that due to the stochastic nature of the model, different runs give slightly different results. Selecting model 2 for this plot option gives similar results. Other models do not perform as well as models 1 and 2 (see Figure S1 for description of models).

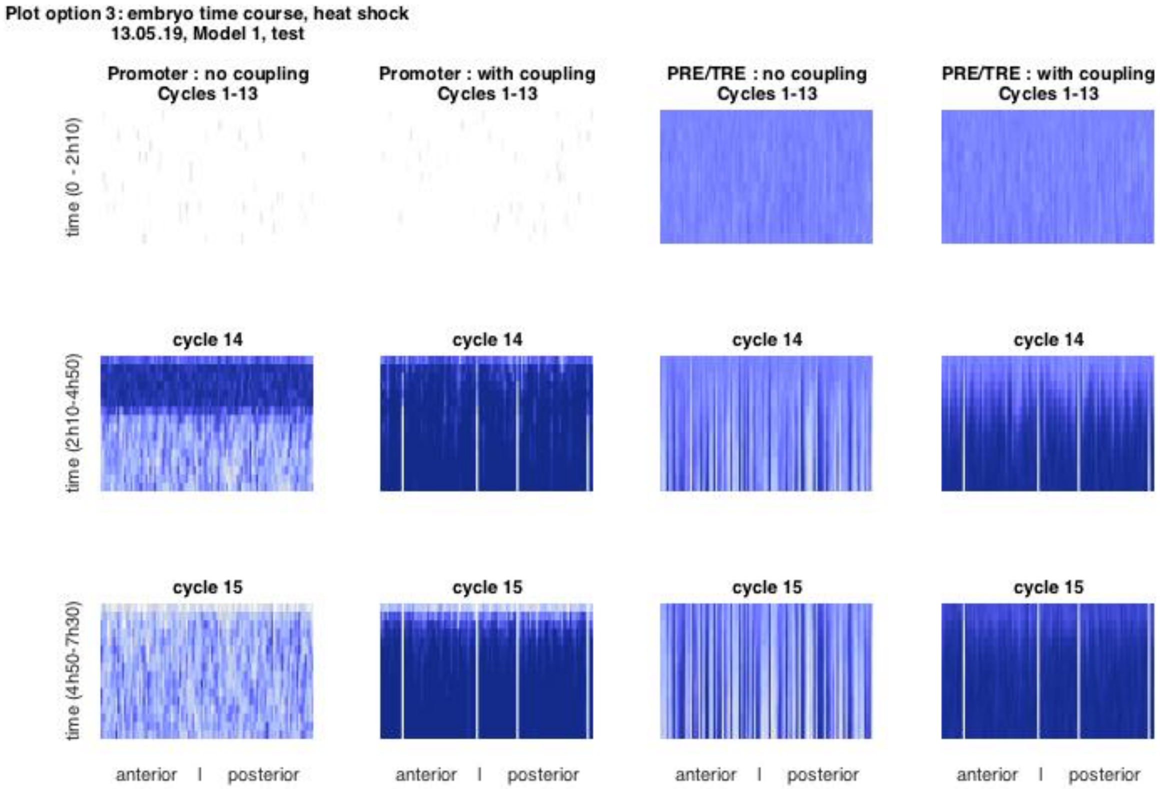

Figure 2. Average state of PRE/TRE and promoter for the last 10 minutes of the simulations shown above, as shown in Figure S3B of the paper. Note that A (anterior) and P (posterior) compartments are shown separately here, whereas they are combined in the paper. Top: Mean and standard deviation across the 50 independent simulations are shown. Bottom: the same data are plotted as box and whisker plots (Matlab default settings, see Figure 2 of Reinig et al for details).

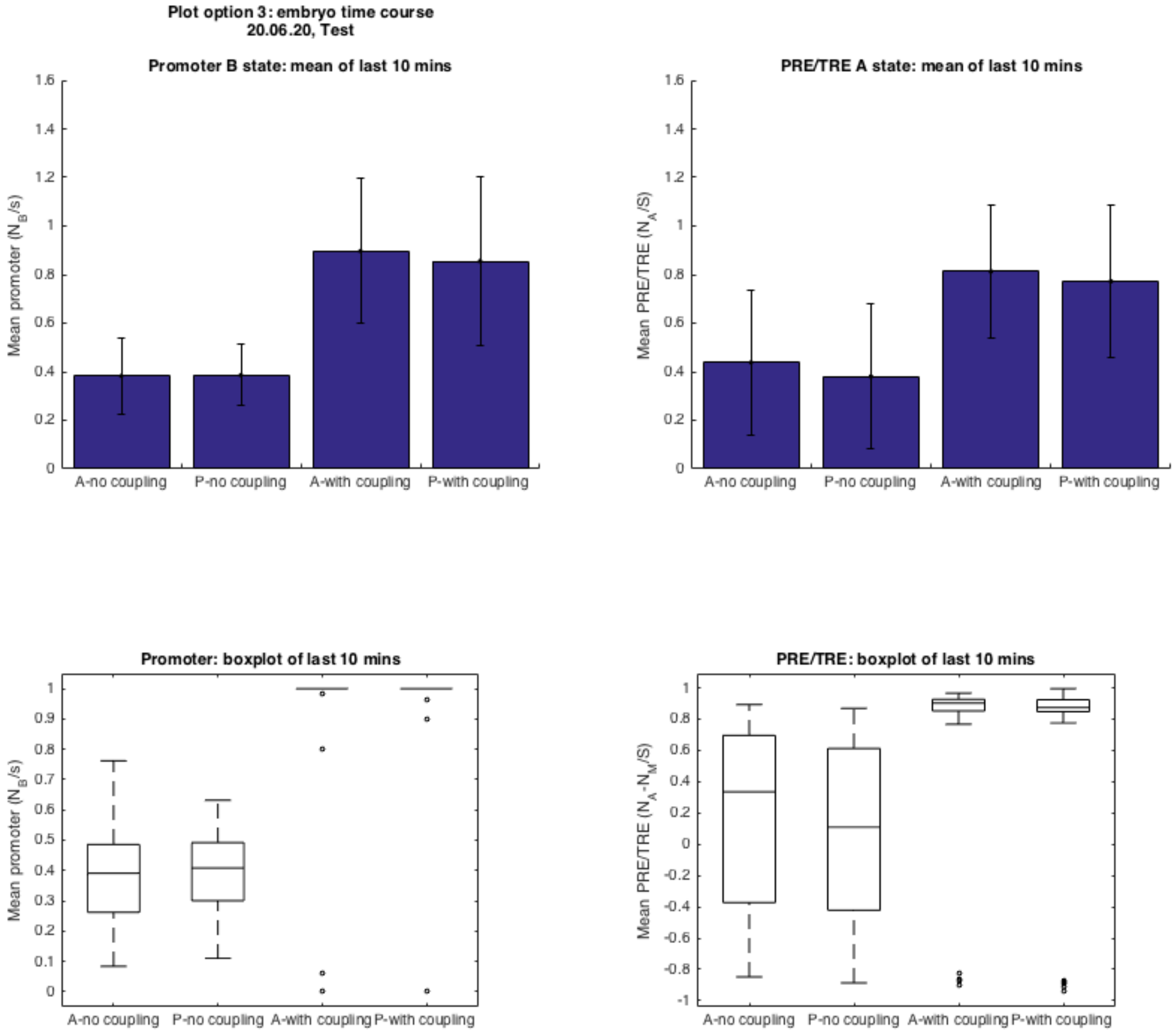

Plot option 4. Runs a parameter scan for the time course simulations shown in Plot option 3, and evaluates memory of heat shocked promoter states.

Inputs:

```
note = '13.05.19, Model 1, test';
plot_option = 4;
model = 1;
n = 100;

Coupling_Strength = 1;
PRE_init = ('U');
Enhancer_init = ('F');

heatshock = 1;
```

Run time ca. 12 mins for n = 100

Input variation:

Model: Other models will give different results as shown in Figure S1G, H.

Coupling_Strength: See Figure S3D. The parameter scan is performed under a single coupling regime. A user- defined coupling strength of 1 defines the parameter $C_{i,m}$ for model 1 as 8, and for model 2 as 4, as shown in figure S3D. Other panels of figure S3D are generated by adjusting Coupling_Strength to 0, 0.25, or 0.5, and/or selecting model 2.

Outputs:

Figure 1. Parameter scan with evaluation of memory of activation as explained in Figure S3C and D of the paper. The plot shows the promoter (left, as shown in Fig S2D) and the PRE/TRE (right, not shown in the paper). Yellow colour indicates good memory. See Fig. S3C for calculation of memory score.

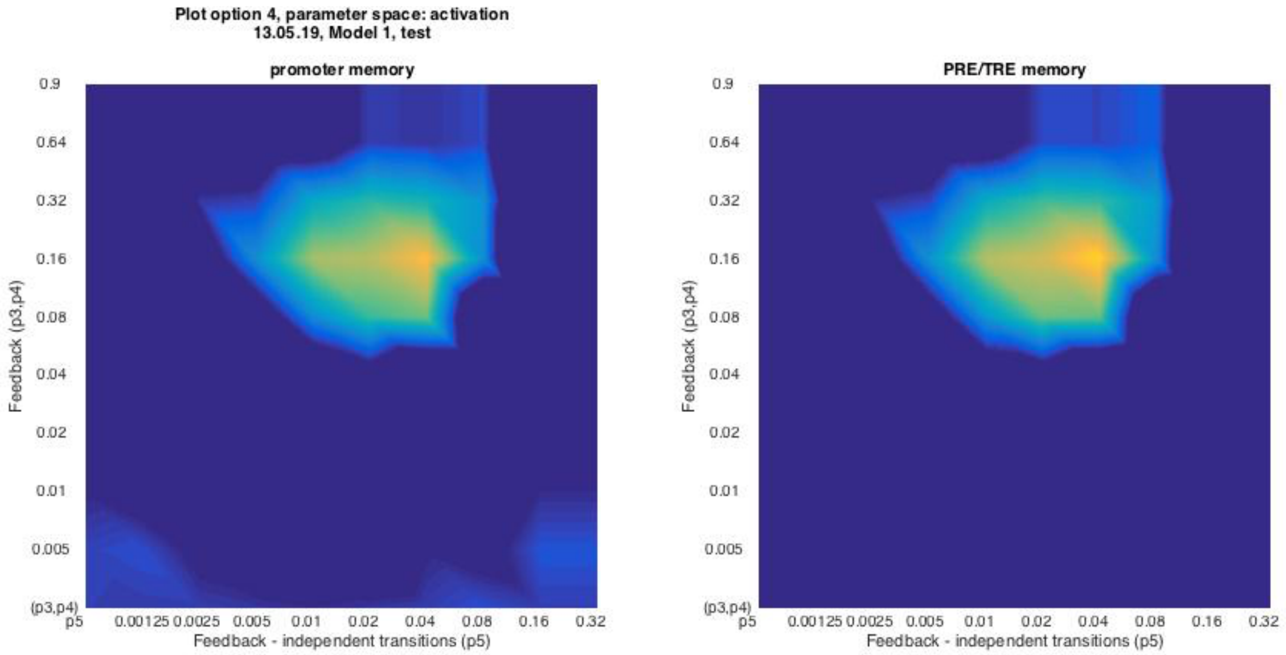

Figure 2. All raw data from parameter scan (not shown in paper). Each plot shows data averaged over last 10 minutes of the phase indicated. “Heatshock”: (3h00- 3h10).“ Maintenance”: (7h20 – 7h30). X-axis shows parameter values in blocks for a given value of parameter 2 (here, p5). Within each block, the values of parameter 1 were varied as indicated below the plots (here (p3,p4)). Pairs of columns represent anterior (left) and posterior (right) of the embryo for each parameter combination, in this example the parameter inputs in anterior and posterior were identical, and difference is due to the stochastic nature of the simulation. Blue indicates active promoter or PRE/TRE. Vertical axis shows 100 repetitions for the same condition.

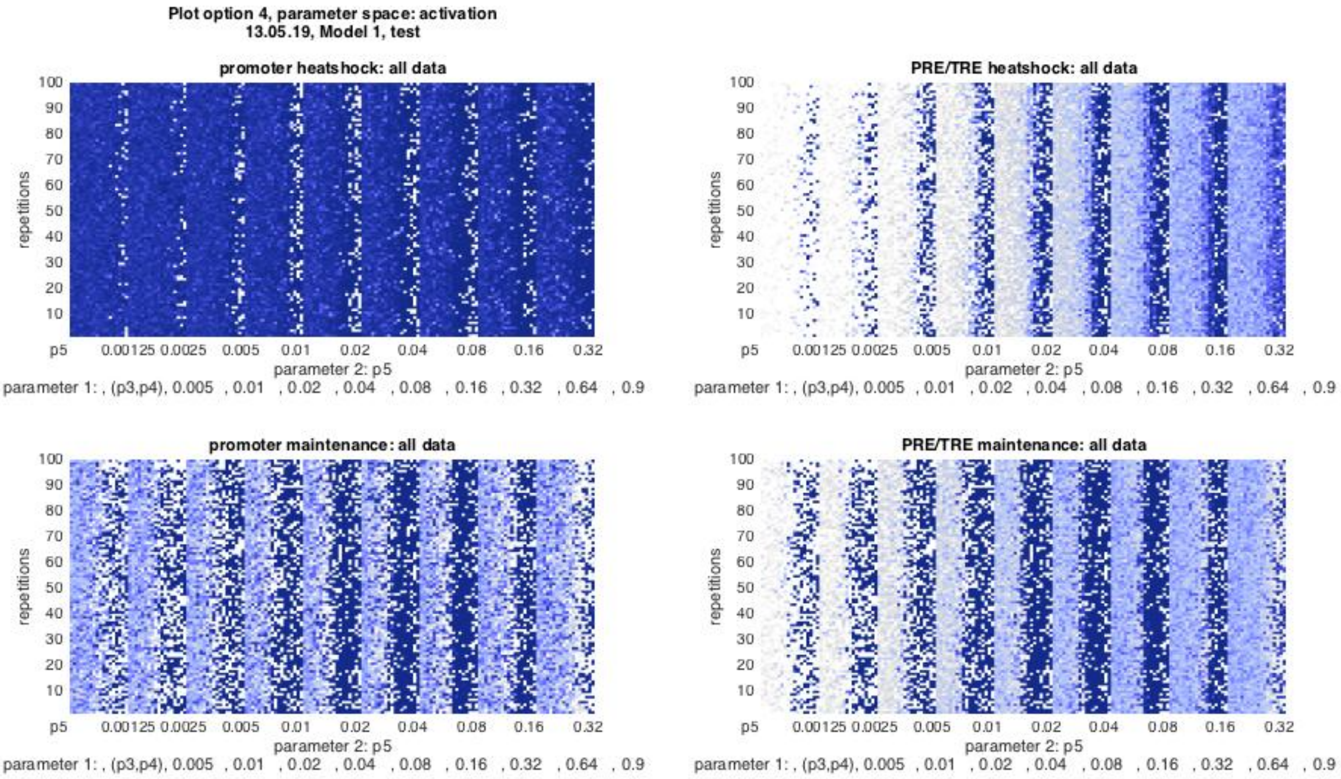

Figure 3. Data from parameter scan averaged over 100 repetitions showing mean and SD. Bottom: Promoter levels averaged across anterior and posterior for each parameter set are shown. Top: promoter memory, calculated as in Fig S3C. High “activation memory” score indicates high maintenance of activation.

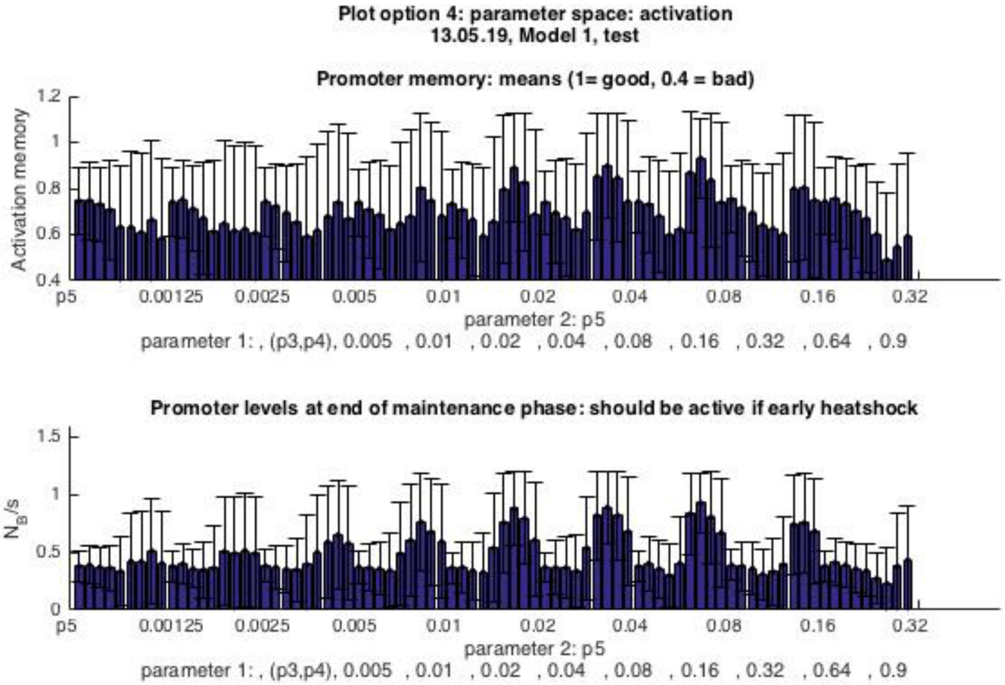

Plot option 5. Runs simulations of eye disc data for *eya* or *bxd* PRE/TREs.

Inputs

```
note = '13.05.19, Model 1, test';
plot_option = 5;
model = 1;
n = 100;

Coupling_Strength = 1;
PRE_init = ('U');
Enhancer_init = ('F');

heatshock = 1;
PRE_ID = ('e');
```

Run time ca. 90 sec for n = 100

Input variation:

n: must be at least 10. The user selects either the *eya* PRE/TRE (PRE_ID = ('e') or the *bxd* PRE/TRE (PRE_ID = ('b'). Outputs for both are shown below.

Output. **Figure 1.** Top. Average profile (n = 100) of promoter and PRE/TRE with and without coupling across the model eye disc. (see Figure 5 and S8 of the paper). Bottom, "Disc" images compiled from 20 rows (see Figure 5, S7 and S8).

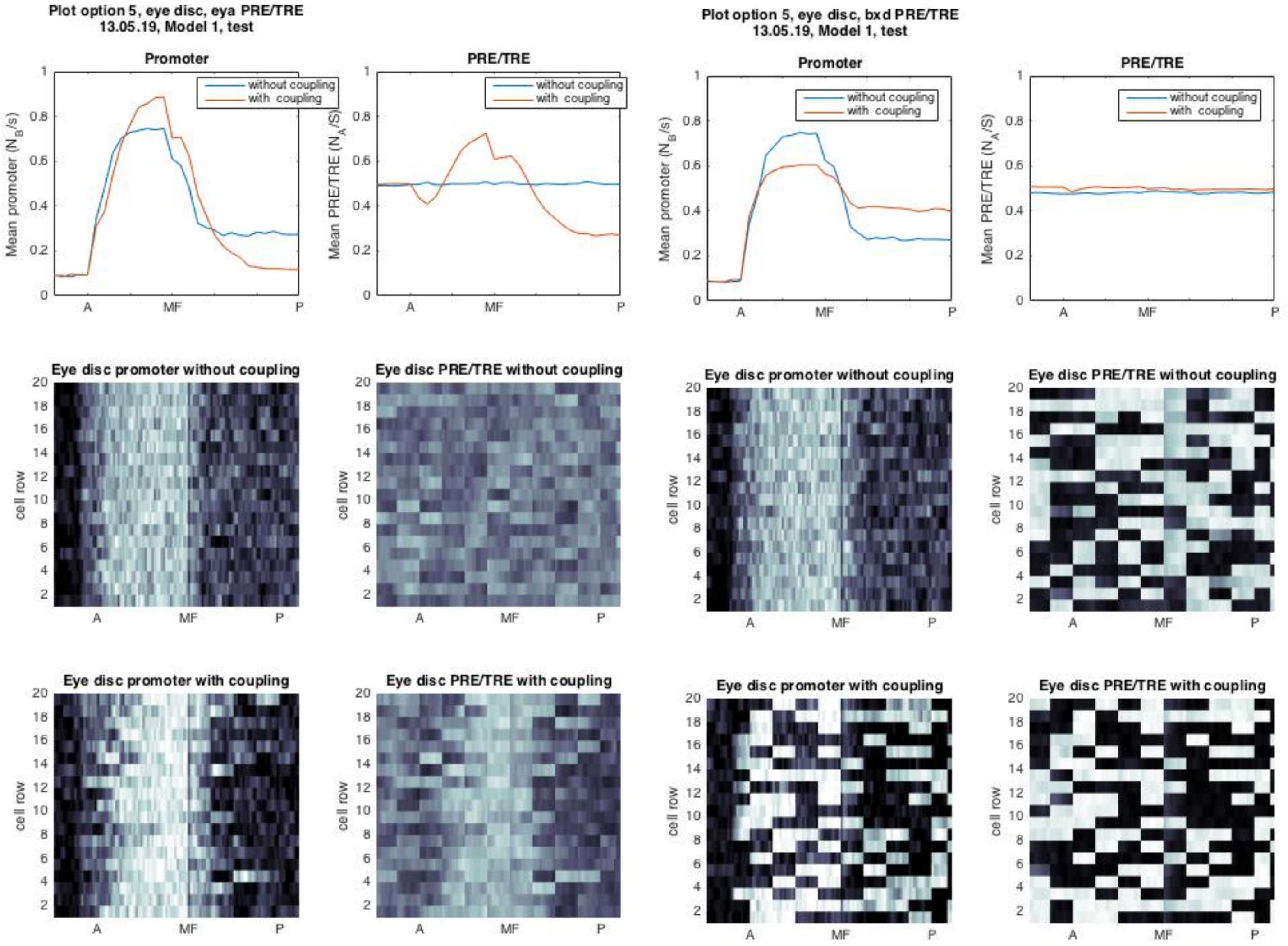

Plot option 6. Runs a parameter scan for the eye disc simulations shown in Plot option 5, and evaluates the fit of the model to data for *eya* and *bxd* PRE/TREs.

Inputs:

```
note = '13.05.19, Model 1, test';
plot_option = 6;
model = 1;
n = 100;
Coupling_Strength = 1;
```

Run time ca. 1 hour for n = 100

Input variation:

Model: Other models will give different results as shown in Figure S1G, H.

Coupling_Strength: See Figure S8. The parameter scan is performed under a single coupling regime. A user- defined coupling strength of 1 defines the parameter $C_{i,m}$ for model 1 as 8, and for model 2 as 4, as shown in figure S8E-G. Other panels of figure S8E-G are generated by adjusting Coupling_Strength to 0, 0.25, or 0.5, and/or selecting model 2.

Output.

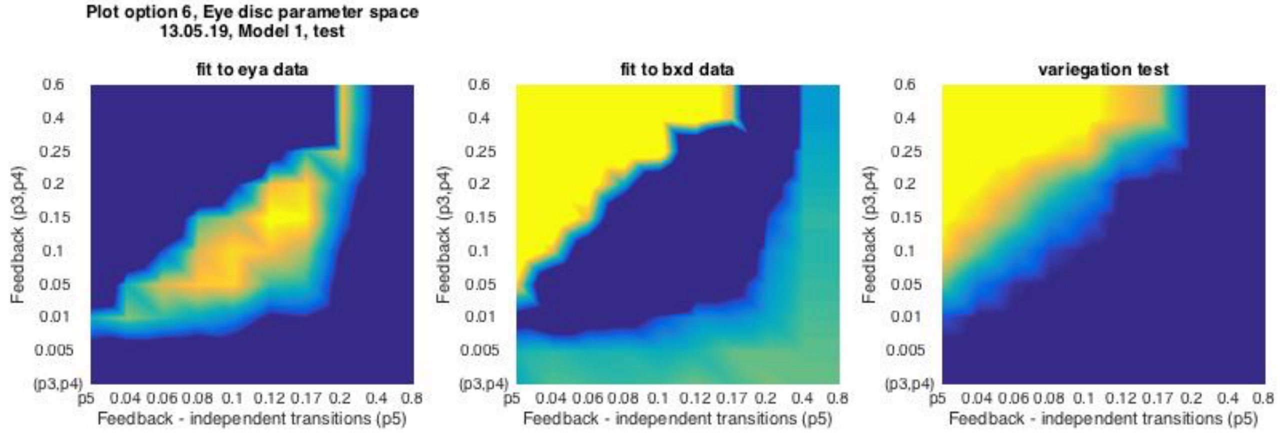

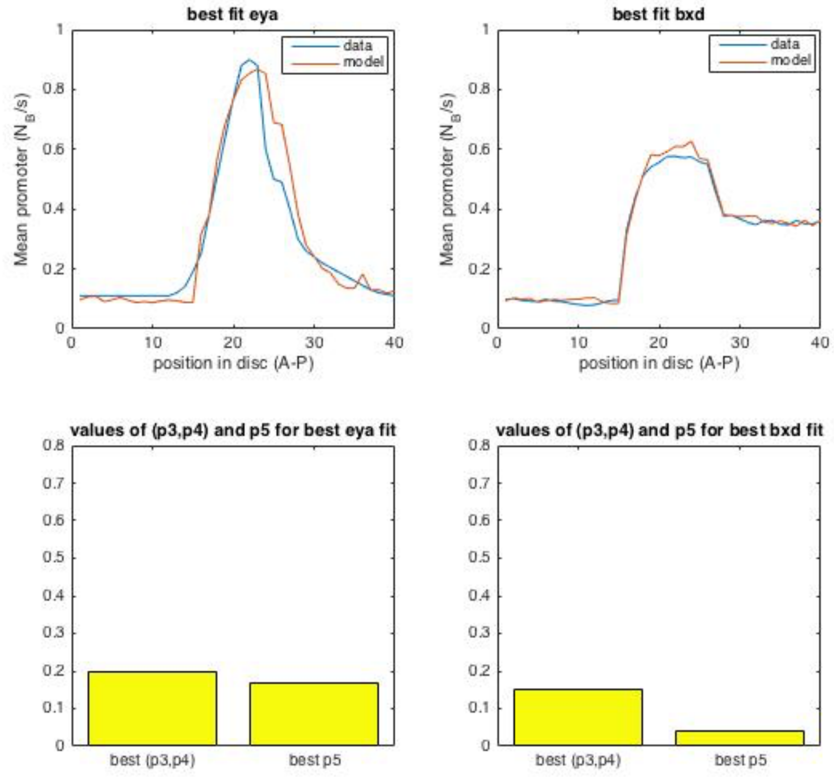

The top three plots show parameter scans. Left, model fit to experimental eya gradient, middle, model fit to bxd experiment. Yellow indicates good fit. Right: Analysis of variegation. Yellow indicates high variegation. (see Figure S8 and legend for explanation of fitting and variegation analysis.

The middle plots show comparison of experimental data (blue curves) and the best fit of the model for eya PRE/TRE and bxd PRE/TRE. Promoter output is shown for data and model with coupling to PRE/TRE. See Figure 5 and S8 for details.

The bottom plots show the parameter values corresponding to the best fits in the figures above. The values are printed in the command window after the run.

Plot option 7. Plots time course for PRE/TRE and promoter during embryogenesis and evaluates states at the end of the run.

Inputs:

```
note = '13.05.19, Model 1, test';
plot_option = 7;.
model = 1;
n = 100;

Coupling_Strength = 1;
PRE_init = ('U');
Enhancer_init = ('F');

heatshock = 1;
PRE_ID = ('b');
```

Run time ca. 15 sec for n = 100

Input variation:

Model: Other models will give different results.
n. Select n = 100 to see average of 100 runs. Select n = 1 to see a single run. Note that pie charts will not work with n-1.
Heatshock. This plot option is linked to the heat shock experiment. The user defines the heat shock timing by entering 0 (no heat shock) 1 (early heat shock, shown here) or 2 (late heat shock).
PRE-ID. The user can enter ‘b’ for *bxd* PRE/TRE (giving memory of early heat shock, as in Reinig et al) or ‘e’ for *eya* PRE, giving interesting variations on this result (not in the paper).

Outputs: **Figure 1.** Time course with and without coupling for PRE/TRE and promoter, averaged over n simulation runs (here, n=100). In this example an early heat shock was given, leading to activation of the PRE/TRE and maintenance of the active promoter (see also Figure 3 in the paper). Mean A or M: Mean A: PRE/TRE proportion of active nucleosomes (N_A/S). Mean M: PRE/TRE proportion of silent nucleosomes (N_M/S). Mean B or F: Mean B: Promoter proportion of bound TF binding sites (N_B/s); Mean F: Promoter proportion of free TF binding sites (N_F/s).

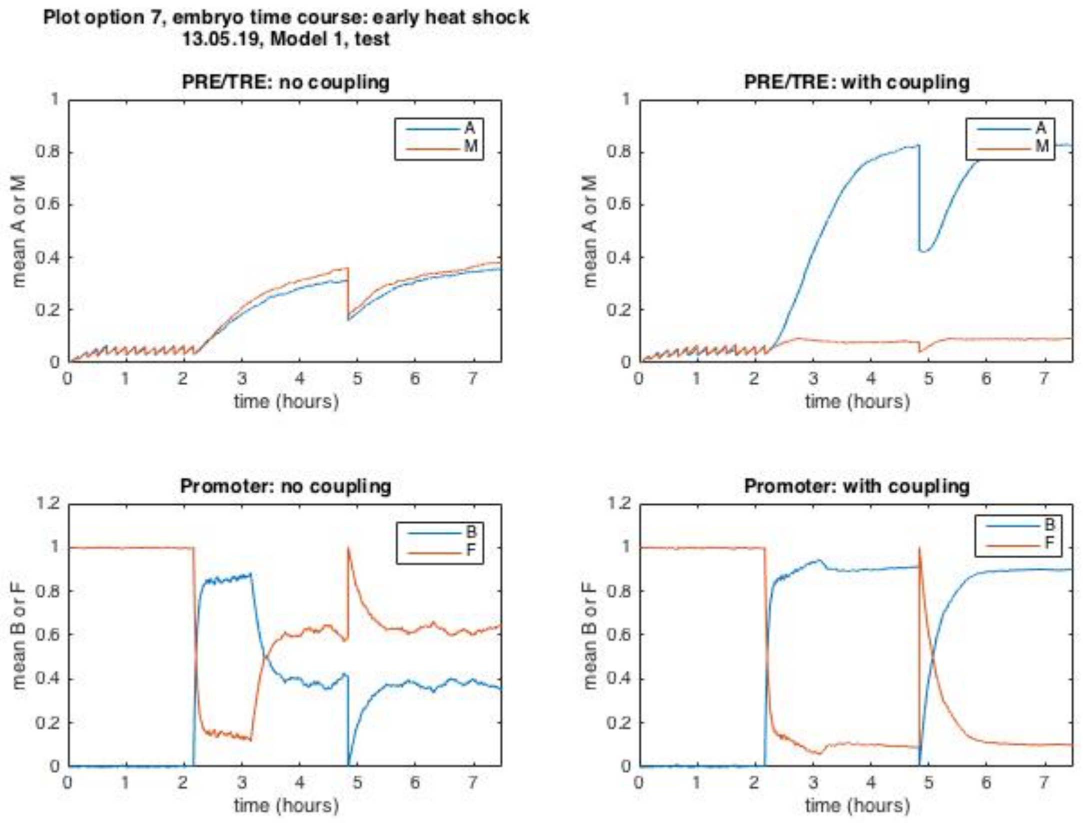

Figure 2. State analysis with and without coupling for PRE/TRE and promoter, averaged over n simulation runs (here, n=100). In this example an early heat shock was given, leading to activation of the PRE/TRE and maintenance of the active promoter (see also Figure 3 in the paper). States are defined as follows: PRE/TRE A: $(N_A/S) > 1.5 * (N_M/S)$. PRE/TRE M: $(N_M/S) > 1.5 * (N_A/S)$. PRE/TRE U: neither of these conditions is fulfilled. Promoter B: $(N_B/s) > 1.5 * (N_F/s)$. Promoter F: $(N_F/s) > 1.5 * (N_B/s)$. Promoter N: neither of these conditions is fulfilled.

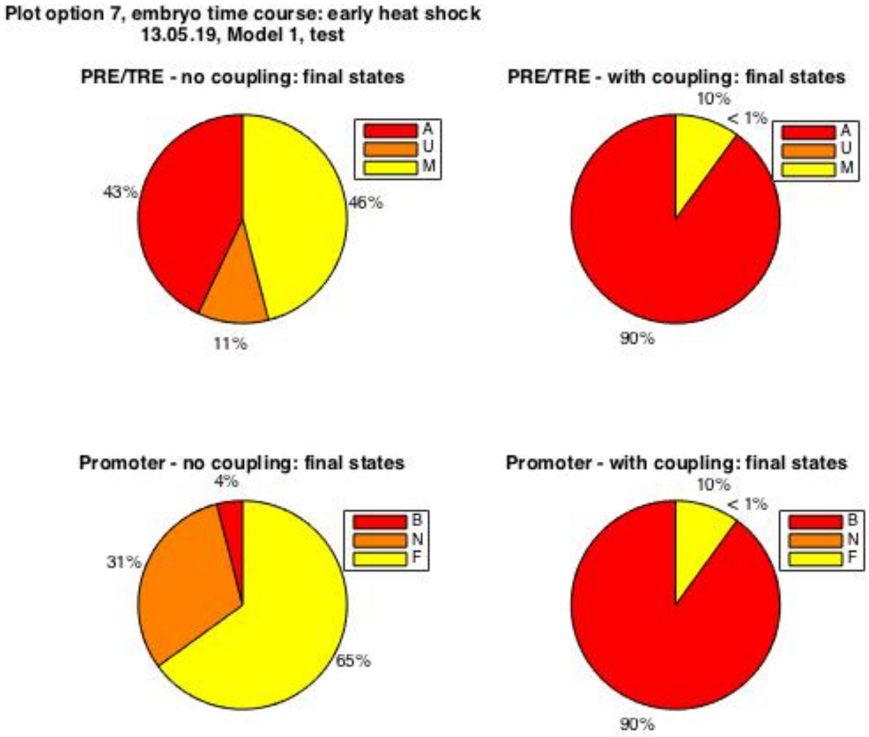

Supplement: Supplementary file 4 — Supplementary Software [file 41467_2020_18507_MOESM4_ESM.zip › Matlab_Reinig_V2_04.07.2020/Reinig_user_manual_04.07.20.pdf]
