# Supplementary material for: A theoretical model of Polycomb/Trithorax action unites stable epigenetic memory and dynamic regulation
Source: Nat Commun. 2020 Sep 22;11:4782. doi: 10.1038/s41467-020-18507-4 (PMC7508846; doi:10.1038/s41467-020-18507-4)
Supplement: Supplementary file 5 — Reporting Summary [file 41467_2020_18507_MOESM5_ESM.pdf]

nature research

Corresponding author(s): Leonie Ringrose

Last updated by author(s): Jul 13, 2020

Reporting Summary

Nature Research wishes to improve the reproducibility of the work that we publish. This form provides structure for consistency and transparency in reporting. For further information on Nature Research policies, see Authors & Referees and the Editorial Policy Checklist .

Statistics

For all statistical analyses, confirm that the following items are present in the figure legend, table legend, main text, or Methods section.

n/a	Confirmed	
☐	☒	The exact sample size (*n*) for each experimental group/condition, given as a discrete number and unit of measurement
☐	☒	A statement on whether measurements were taken from distinct samples or whether the same sample was measured repeatedly
☒	☐	The statistical test(s) used AND whether they are one- or two-sided<br>*Only common tests should be described solely by name; describe more complex techniques in the Methods section.*
☒	☐	A description of all covariates tested
☒	☐	A description of any assumptions or corrections, such as tests of normality and adjustment for multiple comparisons
☒	☐	A full description of the statistical parameters including central tendency (e.g. means) or other basic estimates (e.g. regression coefficient) AND variation (e.g. standard deviation) or associated estimates of uncertainty (e.g. confidence intervals)
☒	☐	For null hypothesis testing, the test statistic (e.g. *F*, *t*, *r*) with confidence intervals, effect sizes, degrees of freedom and *P* value noted<br>*Give P values as exact values whenever suitable.*
☒	☐	For Bayesian analysis, information on the choice of priors and Markov chain Monte Carlo settings
☒	☐	For hierarchical and complex designs, identification of the appropriate level for tests and full reporting of outcomes
☒	☐	Estimates of effect sizes (e.g. Cohen's *d*, Pearson's *r*), indicating how they were calculated

Our web collection on statistics for biologists contains articles on many of the points above.

Software and code

Policy information about availability of computer code

Data collection	None used
Data analysis	Fiji (ImageJ) 2.0.0.-rc_41; ImageJ64, Huygens Essential 16.05, Adobe Photoshop 2-15.01. MetaMorph 7.1.1.0, Image studio Digits 5.2. Custom code written in Matlab R2015b. Deposited in GitHub [https://github.com/Ringrose546/Beyond_memory_V2]

For manuscripts utilizing custom algorithms or software that are central to the research but not yet described in published literature, software must be made available to editors/reviewers. We strongly encourage code deposition in a community repository (e.g. GitHub). See the Nature Research guidelines for submitting code & software for further information.

Data

Policy information about availability of data

All manuscripts must include a data availability statement. This statement should provide the following information, where applicable:

- Accession codes, unique identifiers, or web links for publicly available datasets
- A list of figures that have associated raw data
- A description of any restrictions on data availability

Source data for Figure 4E are provided with this paper. The ChIP-seq data shown in Figure 5A are publically available at [http://compbio.med.harvard.edu/modencode/webpage/Chromatin.v0.6.html#ChIP-seq%20and%20ChIP-chip%20data]. H3K27me3 (track ID 3955), PSC, Posterior Sex Combs (track ID 3960), GAF, GAGA Factor (track ID 4119). The other data sets generated during the current study are available from the corresponding author on reasonable request.

Field-specific reporting

Please select the one below that is the best fit for your research. If you are not sure, read the appropriate sections before making your selection.

☒ Life sciences ☐ Behavioural & social sciences ☐ Ecological, evolutionary & environmental sciences

For a reference copy of the document with all sections, see nature.com/documents/nr-reporting-summary-flat.pdf

Life sciences study design

All studies must disclose on these points even when the disclosure is negative.

Sample size	Relevant for ChIP experiments - two embryo collections, three independent IPs per sample and three qPCR replicates per IP (dilution series) were performed. Larger sample sizes were not feasible due to multiple time point embryo collections. Mean and SD of three IPs (not of PCR replicates) is shown in Fig. 4E. Sample sizes were chosen in line with standard practice in ChIP analysis.
Data exclusions	No data were excluded unless a PCR reaction failed, visible as a missing data point in a dilution series.
Replication	Replication was successful. Three ChIP analyses for each embryo collection, several hundred embryos for each Immunofluorescence slide, 6 to 10 eye imaginal discs for each genotype for in situ hybridisation on transgenic lines, 50 to 100 progeny for each cross for each genotype for genetic experiments, showed similar results.
Randomization	Not relevant because randomisation is used for clinical trials with interventions. We did not perform any such trials.
Blinding	Not relevant because blinding is used for clinical trials to prevent subjects from knowing about their treatment. We did not perform any such trials.

Reporting for specific materials, systems and methods

We require information from authors about some types of materials, experimental systems and methods used in many studies. Here, indicate whether each material, system or method listed is relevant to your study. If you are not sure if a list item applies to your research, read the appropriate section before selecting a response.

Materials & experimental systems

n/a	Involved in the study	
☐	☒	Antibodies
☒	☐	Eukaryotic cell lines
☒	☐	Palaeontology
☐	☒	Animals and other organisms
☒	☐	Human research participants
☒	☐	Clinical data

Methods

n/a	Involved in the study	
☒	☐	ChIP-seq
☒	☐	Flow cytometry
☒	☐	MRI-based neuroimaging

Antibodies

Antibodies used

Primary antibodies : Antibody:Supplier/Catalog no./lot no
Mouse anti Histone H3: Active Motif/39763/5217020, Mab clone 301
Rabbit anti H3K4me1: Abcam/ab8895/gr271478-1
Rabbit anti H3K4me3: Abcam/ab8580/gr240214-3
Rabbit anti H3K27me3: Active Motif/39155/31814017
Rabbit anti H3K36me2: Abcam/ab9049/gr266894-1
Rabbit anti H3K36me3: Abcam/ab9050/gr249065-1
Mouse anti Fluorescein: Roche/11426320/Mab clone 001

Secondary Antibodies
Goat anti Rabbit IgG, Alexa Fluor 555 conjugate: Thermo Fisher Scientific/A21429/1715464
Goat anti Mouse IgG, Alexa Fluor 488 conjugate: Thermo Fisher Scientific/A11029/1705900
Goat anti Rabbit IgG, Horse Radish Peroxidase (HRP) conjugate: Life Technologies/A16104/42-28-042114
Goat anti Mouse IgG, Horse Radish Peroxidase (HRP) conjugate: Life Technologies/A16072/46-137-05115
Goat anti Mouse IgG, Horse Radish Peroxidase (HRP) conjugate: Invitrogen /T-20912

The same information is given in supplementary Tables 4 and 5.

Validation	We performed our own validation of primary antibodies against modified histones presented in Supplementary Figure 4. The mouse anti H3 antibody is validated for ChIP, IF and Western blot on the supplier's website https://www.activemotif.com/catalog/details/39763

Animals and other organisms

Policy information about studies involving animals; ARRIVE guidelines recommended for reporting animal research

Laboratory animals	Drosophila melanogaster OregonR and transgenic derivatives thereof. Males and females.
Wild animals	Not applicable. We used only laboratory strains
Field-collected samples	Not applicable. We used only laboratory strains
Ethics oversight	The work was performed exclusively with Drosophila melanogaster, which does not require ethics documentation according to German law.

Note that full information on the approval of the study protocol must also be provided in the manuscript.
